# Exploring novel mechanistic insights in Alzheimer’s disease by assessing reliability of protein interactions

**DOI:** 10.1038/srep13634

**Published:** 2015-09-08

**Authors:** Ashutosh Malhotra, Erfan Younesi, Sudeep Sahadevan, Joerg Zimmermann, Martin Hofmann-Apitius

**Affiliations:** 1 Department of Bioinformatics, Fraunhofer Institute for Algorithms and Scientific Computing (SCAI), Schloss Birlinghoven, 53754 Sankt Augustin, Germany; 2 European Molecular Biology Laboratory (EMBL), Meyerhofstraße 1, 69117 Heidelberg, Germany; 3 Rheinische Friedrich-Wilhelms-Universität Bonn, Bonn-Aachen International Center for Information Technology, 53113, Bonn, Germany

## Abstract

Protein interaction networks are widely used in computational biology as a graphical means of representing higher-level systemic functions in a computable form. Although, many algorithms exist that seamlessly collect and measure protein interaction information in network models, they often do not provide novel mechanistic insights using quantitative criteria. Measuring information content and knowledge representation in network models about disease mechanisms becomes crucial particularly when exploring new target candidates in a well-defined functional context of a potential disease mechanism. To this end, we have developed a knowledge-based scoring approach that uses literature-derived protein interaction features to quantify protein interaction confidence. Thereby, we introduce the novel concept of knowledge cliffs, regions of the interaction network where a significant gap between high scoring and low scoring interactions is observed, representing a divide between established and emerging knowledge on disease mechanism. To show the application of this approach, we constructed and assessed reliability of a protein-protein interaction model specific to Alzheimer’s disease, which led to screening, and prioritization of four novel protein candidates. Evaluation of the identified candidates showed that two of them are already followed in clinical trials for testing potential AD drugs.

Protein function is usually governed by protein-protein interactions (PPIs); therefore, defining the interaction partners of proteins may help to understand their biological role. Particularly, for complex idiopathic diseases like Alzheimer’s disease (AD), the limited efficacy of available treatments^1^ indicates a strong need for deciphering alternative disease mechanisms. Protein-protein interaction (PPI) networks are popular means to obtain biological insights into disease mechanism^2^. The prospect of deducing further conclusions and speculations makes PPI data interesting and important. Many research groups are progressively working in this domain and thus, an ever-increasing amount of data on the interaction of proteins is being produced. As a consequence, there is also substantial interest in developing a rationale for determination of the reliability of protein-protein interactions.

Protein-protein interactions can be scored differently based on various parameters. For instance, they can be scored based on structural information, network topology, experimental conditions, literature confidence, similarity of the functional annotations for interactors, or evolutionary conservation^3^. Therefore combining all these parameters into a single, central scoring schema appears to be a daunting task. Depending on the final objective and information resources, different frameworks and scoring systems exist that can uniquely measure the confidence of protein interactions. For instance, PSISCORE^3^ is a framework for qualitative assessment of molecular interaction data that makes use of multiple confidence scoring servers to provide and compare scores on interaction data. Similarly, MINT database^4^ uses a scoring schema based on heuristic integration of the experimental evidence, number of studies, orthology and community trust to calculate a score called MINT score^4^. HIPPIE (Human Integrated Protein-Protein Interaction reference)^5^, is another scoring system that uses experimental confidence as a prime feature for interaction reliability whereas String database^6^ has its own scoring schema that determines interaction validity by taking into account factors such as experimental evidence, neighborhood, Co-occurrence, Co-expression and number of publications supporting interaction. One disadvantage, however, that is common to all of these scoring functions is that they do not consider negative interactions or non-interacting proteins unequivocally. Statements about negative interactions in the literature are very rare, although approximations have been proposed^7^. Also, apart from some recent efforts^8^, most of the above-mentioned strategies just consider assigning score to an interaction without demonstrating the true value that comes with the confidence assessment. In this work, we show, how an assessment of the knowledge quality or the confidence behind a protein interaction network representing a disease mechanism, can serve as a rational to assist decision making for emerging targets and the selection of biomarker candidates. In the present work, we introduce a novel scoring strategy, which incorporates knowledge quality measurement parameters into protein interaction networks in a way that enables us to reliably distinguish established (well known) from emerging (speculative or surging) knowledge. This is particularly useful for investigating molecular mechanisms underlying complex and idiopathic diseases such as AD where the focus on integrating both established and emerging knowledge into integrative models is more likely to generate novel insights rather than considering only established, well-known information. Hence, our aim was to find and prioritize emerging knowledge niches within the AD domain that have the potential to add value to the ongoing research activities.

The core of our workflow encompasses empirically optimized guidelines based on multiple criteria decision-making (MCDM) method^9^. We show that application of this methodology to AD-specific PPIs, in the first place, provides further support for the existing disease targets or known, valid, targeted interactions. Moreover, this assessment highlights regions of the interaction network where high-score interactions are in vicinity to low-score interactions, thus leading to a sudden decline of score. We define such suburbs in a PPI network as “Knowledge Cliffs”, i.e. coherent sub-graphs in which the difference of adjoining interaction scores is greater than a defined threshold. Investigating such Knowledge Cliff regions help us identify new emerging candidates in neighborhood to the “well-known” proteins, which could serve as upstream causal entity with a relevant downstream bio-clinical effect on an established target. We aimed to obtain novel mechanistic insights into possible disease processes through such candidates as they are already embedded in a well-defined functional context. Our work demonstrates, how mechanistic modelling and identification of reliable and novel findings in a network context can increase chances to identify new targets causally involved in the disease mechanism.

## Methodology

### Building literature-derived Alzheimer’s-specific PPI network

We have built a reliable and curated Alzheimer’s-specific PPI network using the biomedical literature, which is arguably one of the most comprehensive information resources. The Alzheimer’s-specific protein interaction network has been constructed by mining AD-specific statements in the literature (PubMed abstracts + PubMed central documents). Initially, a supervised machine learning approach designed by Bobic *et al.*^10^ was used to automatically extract sentences from the literature relevant to AD where two proteins have been reported to interact with each other. Such evidence were further manually filtered by 3 annotators following strict annotation guidelines (Supplementary file 1). Annotators were looking typically for relationships existing in the literature where two proteins have been shown to interact physically with each other under AD conditions. These annotators individually annotated each and every protein interaction evidence and their annotations were used to calculate the inter-annotator agreement (IAA)^11^. The IAA determines the quality and acceptability of annotations among annotators and provides a rationale for measuring the quality of introduced annotation guidelines. The kappa value between the annotators was calculated as high as 0.81, which indicates an acceptable agreement, given the complicated nature of the annotated evidence lines.

Although, curated PPI information can be extracted from publicly available databases like STRING (http://string-db.org) where the source of interaction information comes from multiple methodologies^6^ or MINT (http://mint.bio.uniroma2.it/mint/Welcome.do) where the source of interaction information is full-text publications^4^, this information is not context specific (i.e. exclusively mentioning interactions taking place during AD). Also, the number of PPIs in databases vary widely from as low as 100 to over 36,000 interactions and overlap of PPIs even within the same category of databases (e.g. between literature-derived databases) is low^12^,^13^, with rare exception shared by databases such as MINT, DIP, IntAct that are coordinated under IMEx collaboration framework (http://www.imexconsortium.org)^14^ to intentionally avoid overlap. Hence, we directly mined the literature to derive PPI information in the context of molecular events underlying AD.

Interactions (edges) of the derived AD network are directed and annotated with entities like type_of_experiment_performed (which can have two values: *InVivo* or *InVitro*) to confirm an interaction. All different parameters annotated by annotators corresponding to a PPI have been mentioned in details in the annotation guidelines (Supplementary file 1). These guidelines have been prepared after input and feedback from several domain experts in the field (see Acknowledgments). The Cytoscape environment^15^ was finally used for the visualization of the AD specific PPI network.

### Measuring reliability of protein interactions

Calculation of the reliability score assigned to each edge in the network takes into account the following attributes extracted from the literature:

- Number of independent evidence in the literature in support of PPIs.
- Number of contradictory evidence.
- Experiment type: *In-vivo* (human/mouse/cell lines), *in-vitro* (using physicochemical methods (rated as the most reliable by domain experts), genetic methods or library-based methods (rated as the least reliable by domain experts).
- Factual or Hypothetical statement backing up a claim (as inferred by the annotators).
- Community trust of the publication reporting PPI.

Next important aspect for confidence measurement concerns combination and weighting of these attributes. Application of machine learning techniques for weight assignment in this particular task was not an option due to lack of high quality data for training and testing (particularly data supporting PPI using Human/Mouse models and the very limited availability of sufficient contradictory information or negative data). Hence, the core of the developed workflow encompasses empirically optimized guidelines based on multiple criteria decision making (MCDM)^10^. This discipline of statistical analysis explicitly considers multiple criteria in decision-making environments. To adhere to requirements of MCDM, each of the above-mentioned attributes has been flattened into 12 categories or factors. Every evidence (supportive or contradictory) backing up or contradicting a protein-protein interaction was classified in one of these 12 categories (see Supplementary file 2 for further details).

Further, we asked experts in the AD field (clinicians, molecular biologists, proteomics experts) to rank multiple, often conflicting, factors based on which they judge the reliability of a PPI, with rank “1” assigned to the most reliable factor and “12” to the least reliable one (Supplementary dataset 1). Proportionally, ranks supplied by the decision makers (experts) were converted into weights using the “rank sum linear weight” method^9^,^15^, which has been shown to outperform other methods for the defined factor of choice^16^.


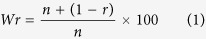


Where *Wr* is the final weight, *r* is the factor rank and *n* is the total number of ranked factors.

Derived weight for each factor along with the total count of supportive and contradictory evidence forms the basis for the designed scoring function. In addition, the calculation of scores also takes into account a factor representing the community confidence in a finding, i.e. the citation-based impact factor. We are fully aware of the potentially strong bias underlying impact factor and there is good reason why impact factors are discussed controversially. However, given pros and cons, and particularly, after feedback from several domain experts on attribute selection and ranking, we found that most experimental biologists and clinical researchers actually acknowledge the usefulness of impact factor in assessing the confidence especially when it comes to literature-derived data. Hence, a list of impact factors derived from 932 biological journals (manually filtered) were discretized into 12 ranks using a quintile-based distribution and finally following the above defined strategy these ranks were converted into weights.

### PPI confidence scoring function

The score (*S*) was calculated as a weighted sum of three different factors including *Evidence,* a function of the number of studies in which an interaction was detected or not detected; *Weight of the bin,* a function which combines quality of experimental techniques (as ranked by experts) along with the reliability expressed in textual evidence provided by the authors (Hypothesis/Fact); and *Community confidence,* a function measuring the impact that individual articles published in particular journal have on research community. Each of these factors was combined in the following formula to calculate the score for measuring reliability of a particular protein interaction.


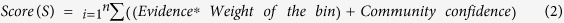


where i = 1 is the default value assigned to every new evidence and n is the total number of evidence lines backing up a particular protein-protein interaction. Evidence can have a positive or negative value depending on whether it is a supporting evidence or a contradiction. Since we aimed at converting the score *(S)* into a normalized value that could easily differentiate between the highly studied (established) interactions and the less studied (emerging) interactions, the final scores were normalized into a 0-1 scale. Protein interactions with a score close to 1 represent most studied interactions (frequently reported in the literature) and interactions with a score near to 0 are the ones with least confidence or less studied interactions. As a first step for score normalization, we log transformed the calculated score (*S*). This log-transformed score was referred to as log_score. This step was necessary as our final scores were highly skewed towards the most studied interactions. In the next step, we normalized the log_transformed_ final_score into a 0-1 scale using the equation:


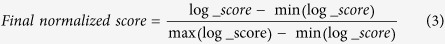


An R script has been generated which takes the PPI annotated file as input and calculates the “Final normalized score” using the above-mentioned formulas (Supplementary Dataset 2).

### Evaluation of the scored network model

Typically, it is very difficult to judge about the completeness of a network because of the varying scales of information available from different resources that can be integrated into the network. However, such constrains can be partly mitigated if one tries to judge the completeness of information from the literature. In order to evaluate the completeness of the presented AD network (Supplementary file 3), we performed an enrichment analysis using the relation mining approach^11^: we generated a “test-corpus” of 200 randomly selected full text articles from PubMed containing information relevant to PPIs in AD. Three new independent annotators manually crosschecked the test-corpus and mined for AD-specific PPI evidence. Finally, we compared the network coming from the test-corpus (Supplementary file 4) to our previously built AD network. We found a complete overlap conceivably showing that our AD network is complete as per the AD related PPI information existing in the literature.

In another scenario, we mined protein candidates reported as biomarkers for AD in the literature using a biomarker terminology^17^ within our text mining system SCAIView^18^. The biomarker terminology helped us to systematically look for evidence lines (apart from the keyword biomarker) in the literature where a particular candidate has been shown to be differentially expressed or have been proven as a genetic marker for AD. This approach - along with manual curation - resulted in a collection of statements describing role of a particular candidate as a biomarker (Supplementary file 5). An overlay of 43 literature-derived putative AD biomarkers on our AD-network resulted in a 100% superimpose (Supplementary file 6) showing that this network is highly enriched for the presence of the majority of reportedly altered proteins under AD condition. Also, the more established biomarker candidates (e.g. APP, MAPT, APOE, PSEN1) were present as hub genes in our network.

### Network analysis and cause-and-effect knowledge model generation

In an attempt to highlight network regions linking established and emerging knowledge in the defined AD context, we looked for neighboring interactions with highly varying interaction scores (see Results). Ultimately, we aimed to find new emerging candidates neighboring to the “well-known” proteins, which could serve as upstream causal entity with a relevant downstream bio-clinical effect on an established target. Further, to discover the potential mechanism underlying the role and functional involvement of a putative candidate, we tried to embed the candidate into a model representing causal and correlative relationships. This embedding in a bigger context further helps in rationalizing candidate selection and generation of hypotheses on putative disease mechanisms.

**Figure 1:**
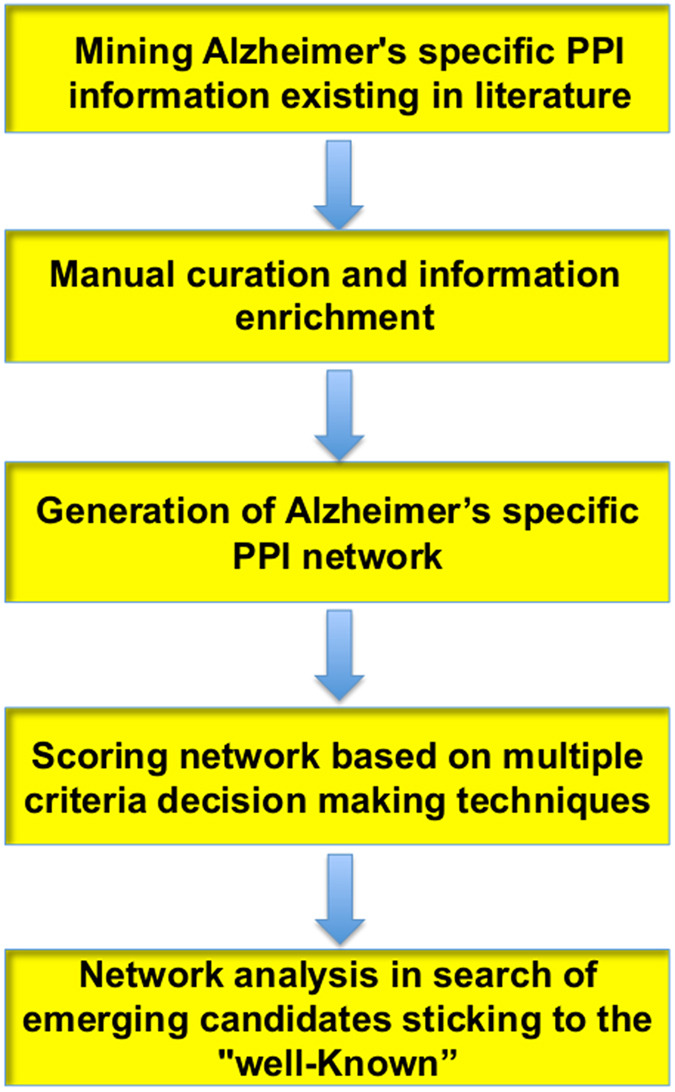
Workflow of the methodology developed to identify new emerging AD candidates. The workflow shows the steps used for the construction of a confidence assessed Alzheimer specific protein-protein interaction network that was further used to filter a set of emerging potential AD candidates existing in the network.

An overview of the methodology described in this section is provided in Fig. 1.

## Results

### Construction of manually curated AD-specific PPI network

We have constructed a directed protein interaction network specific to Alzheimer’s disease, which consists of 301 nodes (proteins) and 339 edges (protein interactions) (Supplementary file 3). By this means, we tried to model physical protein-protein interactions in the AD context based on literature reports and estimate the PPI confidence based on features described in the methodology section. Using this network, we aimed to identify key proteins and protein interactions (established and emerging) involved in molecular changes underlying AD.

### Validation of the scored network model

For establishing the relevance, we made a context-free comparison of the top 5 high scoring interactions as predicted by our methodology with other scoring functions e.g. String^7^, and HIPPIE^6^. Such test frames allow for analyzing the uniformity of scores with available benchmarks such as prior knowledge. We observed an alignment in results when our scoring output was compared to other scoring results. Top scored interactions predicted by using our approach were scored high by other scoring schemes too. Additionally, our top scored interaction (BACE1-APP) has been the most targeted interaction^19^ for the development of inhibitor drugs to treat Alzheimer’s disease^20^,^21^, providing further translational validation for our scoring function.

### Analysis of scored Alzheimer’s disease network

**Figure 2:**
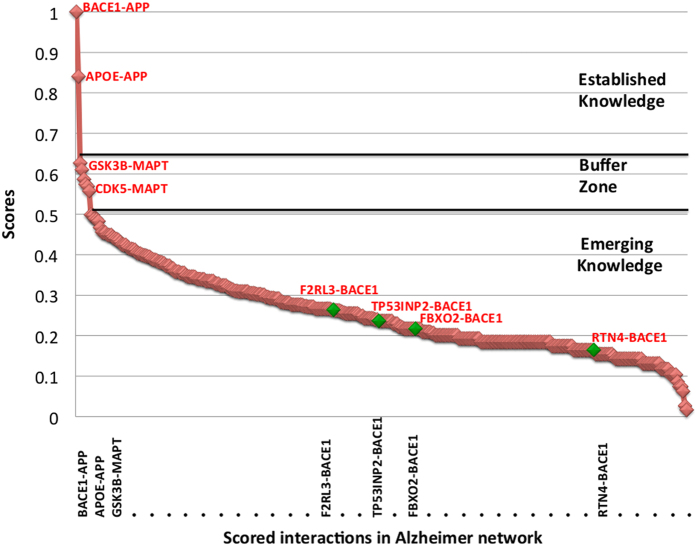
Distribution of interaction scores. Each interaction has been assigned a score between 0-1 based upon assessment of the literature confidence. Score distribution segments the interactions into three sections: Established knowledge (High scoring interactions), Buffer zone (interactions with scores between established and emerging), Emerging knowledge (Low scoring interactions).

The scored network provides a good resolution at the level of protein interactions based on which research trends can be analyzed and discussed. Figure 2 shows the distribution of scores over AD-specific protein interactions, varying from highly scored interactions to low scoring ones. However, a low score does not rule out the possibility of interaction. It is just that there is only limited experimental data and lines of evidence available for that particular interaction. Also notable is the clear distribution of scores (Fig. 2) in three segments, namely “established knowledge zone”, “buffer zone”, and “emerging knowledge zone” that categorize protein interactions based on the reliability assessment.

Our scoring approach assigned top score to APP-BACE1 interaction, consistent with the popularity (how often a particular interaction has been studied in the literature) of the interaction in the context of AD. We found 145 studies in the literature that confirm BACE1 interacts with APP in AD patients, making it the most established and well-studied interaction. Beside BACE1-APP, abundant evidence also exists in the literature for APOE-APP interaction amounting to a high score of 0.84. Accordingly, we consider these top scored interactions as “established knowledge” or most notable interactions in the AD domain. Other top scoring interactions following the above-described interactions include GSK3B-MAPT, CDK5-MAPT, and PSEN1-APP. Although these interactions are also well studied, yet the number of independent supporting studies for them is not as high as number studies supporting BACE1-APP and APOE-APP interactions. Hence, we observed a rapid decrease in scores for these interactions.

We scored the reliability of every protein pair in the AD network and it was clear that high-ranking interactions have been extensively studied for their role in the disease. Nonetheless, we also observed that the majority of AD-specific PPIs got a low score (below 0.5). We did not find many independent studies in the literature in support of these PPIs as these represent emerging knowledge and could be seen as opportunities for researchers to provide new contributions. In particular, among these low-scoring interactions, we found valid pairs embedded in the defined functional context that are likely to be involved in the potential disease mechanism and certainly worth further investigation. Subsequent exploration of such emerging knowledge niches can provide added value to ongoing research activities. Hence, we took advantage of the directed scored network edges to find such candidates in our AD network.

### Neighboring high and low score regions: Knowledge cliff

**Figure 3:**
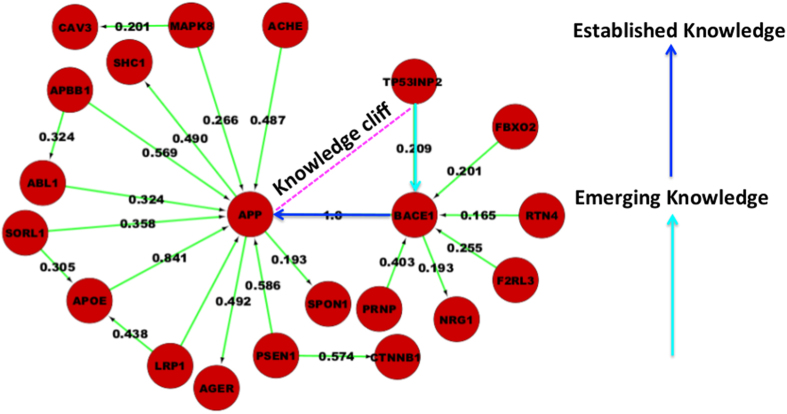
Knowledge cliff representation with in a disease network. Coherent sub-graphs where ratio of adjoining interaction scores is maximum are represented as knowledge cliff. Knowledge cliff helps us finding new emerging candidates sticking to the established candidates, which are well known to play a role in the disease.

Assessing the confidence of AD-specific PPIs provided further support for the existing disease targets or known valid targeted interactions. For instance, APP-BACE1 interaction was scored highest in the network. Besides, these assessment points towards regions with high score interactions, which form a coherent (uni-directional) sub graph^22^ and introduce a sudden decline of score forms “Knowledge Cliffs” (Fig. 3).

For a weighted network or graph represented as G = (V, E) where V represents the vertices of the graph and E represents edges connecting these vertices.

Knowledge cliffs within a weighted graph (G) are sub-graphs (S) forming a unidirectional chain (i->j->k), where i, j, k are vertices of graph G satisfying the conditions W_IJ_>0 and W_JK_ >0. Additionally, the difference between W_IJ_ and W_JK_ has to be greater than a predefined threshold (c): (W_IJ_- W_JK_) > = c.

The value of chosen threshold (c) can vary between 0.1 to 0.99 and can be chosen as per the usage scenario. In this study, the goal is to find new emerging candidates neighboring to the most “well-known” proteins involved in AD, hence a high threshold value(c) = 0.75 has been chosen to identify relevant knowledge cliffs existing within the scored AD network. With the defined threshold choice of 0.75, we found 4 knowledge cliffs in our AD network.

Targeting such Knowledge Cliff regions help us find new emerging candidates neighboring to the “well-known” proteins. We aimed to derive all such candidates in a systematic way as they are already embedded in a disease-specific functional context. In this work, we focused on knowledge cliffs next to some of the most-established interactions such as BACE1-APP and found four “potential new candidates”, namely TP53INP2, RTN4, F2RL3, and FBXO2, presumably new targets for Alzheimer’s disease as guided by the knowledge cliff concept.

### Statistical validation of knowledge cliffs within a network

We aim to statistically validate knowledge cliffs identified within the network against a null hypothesis of random occurrence of edges. In order to accomplish this, we employed a “permutation test” based on the assumption of an independence of the edges. Here we generated 10,000 random networks preserving the node degree distribution in the original Alzheimer’s network. From these 10,000 random networks, the probability of occurrence of an edge (p-value of the edge in the original network) at random network was calculated (Supplementary file 7). The probability of a knowledge cliff occurring at random (p-value of the knowledge cliff) was calculated as the product of all edge probabilities in the knowledge cliff. Based on a p-value threshold (p < 0.05), all knowledge cliffs identified in our network were found to be statistically significant (Supplementary file 7).

### Interpretation of mechanistic causal models

**Figure 4:**
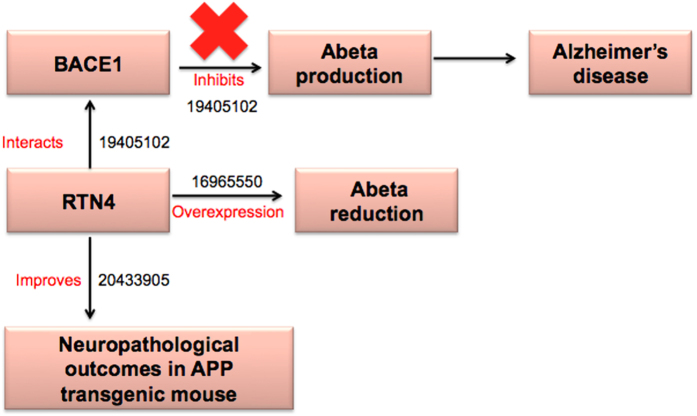
Cartoon-like representations of putative candidate: Reticulon 4 (RTN4). The model illustrates the functional role of the proposed candidate at a mechanistic level in Alzheimer’s disease supported by PubMed identifiers at edges.

To show the relevance of the pinpointed candidates to AD, we modelled their functional context by integrating literature-derived ‘cause and effect’ relationships into cause-and-effect knowledge models. Such models allow for enhanced contextual reasoning at a higher resolution so that modelling the “chains of causal relationships” around putative candidates can provide new insights into their potential mechanistic involvement in the disease. For instance, the RTN4 model (Fig. 4) shows that RTN4 interacts with BACE1 and inhibits its ability to produce Abeta^23^. Also, increased expression of RTN4 results in reduced secretion of Abeta^24^ and there is evidence that RTN4 improves neuro-pathological outcomes such as improved learning and memory deficits in APP transgenic mice^25^. These arrays of evidence lend support to the notion of a putative role for RTN4 in pathology of AD and provide hints about the potential use of RTN4 as a new drug target for AD. This idea is further supported by the finding that Bexarotene^26^- an FDA approved anti-cancer agent, acts as an RTN4 agonist and has been shown to reduce Abeta in AD mouse brain^27^. Currently, there is already a clinical trial (www.clinicalTrials.gov ID: NCT01782742) going on to test if Bexarotene can be used as a drug for treating AD.

**Figure 5:**
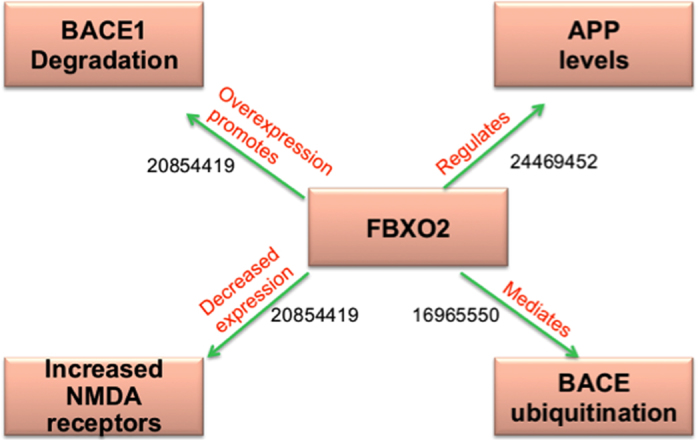
Cartoon-like representations of putative candidate: F-box protein 2 (FBXO2). The model illustrates the functional role of the proposed candidate at a mechanistic level in Alzheimer’s disease supported by PubMed identifiers at edges.

Similarly, we reasoned over interlinked molecules and processes around FBXO2 by combining evidence scattered in the literature (Fig. 5). Accordingly, increased expression of FBXO2 has been shown to decrease BACE1 protein levels and activity^28^, which in turn, leads to reduced beta-amyloid levels and rescue of synaptic deficits in an AD mouse model. Additionally, recent evidence from Atkin *et al.* (2014) suggest that APP is itself a substrate for FBXO2 and APP levels were decreased in the presence of FBXO2 in non-neuronal cells, and increased in both cultured hippocampal neurons and brain tissue from FBXO2 knockout mice^29^. Loss of FBXO2 also has been shown to result in greater expression and surface localization of NMDA receptors^29^, which play a role in the AD pathogenesis.

**Figure 6:**
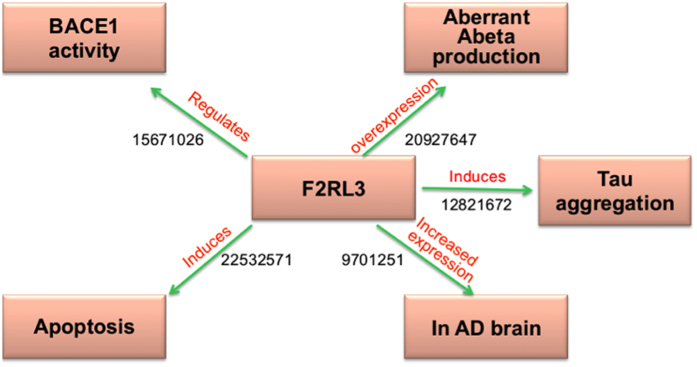
Cartoon-like representations of putative candidate: Thrombin Receptor-Like 3 (F2RL3). The model illustrates the functional role of the proposed candidate at a mechanistic level in Alzheimer’s disease supported by PubMed identifiers at edges.

Modification of the apoptotic cascade could be considered as a primary therapeutic strategy for AD, as implied by our models. Accordingly, our approach was able to detect another candidate, the F2RL3 protein, which induces apoptosis^30^ and may contribute to pathology of AD (Fig. 6). F2RL3 has been known for its involvement in the regulation of beta-secretase cleavage of Alzheimer’s amyloid precursor protein^31^ and its overexpression is accompanied by aberrant Abeta production^32^. F2RL3 has been predicted to induce tau hyper- phosphorylation^33^. All this knowledge may be instrumental for considering F2RL3 as a potential target candidate in AD.

**Figure 7:**
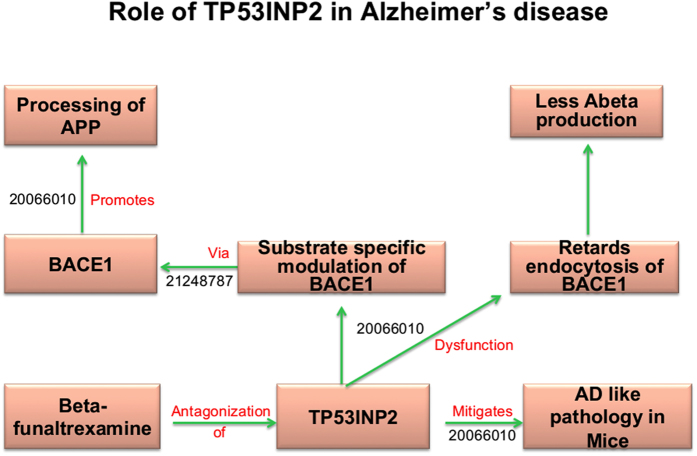
Cartoon-like representations of putative candidate: Tumor Protein P53 Inducible Nuclear Protein (TP53INP2). The model illustrates the functional role of the proposed candidate at a mechanistic level in Alzheimer’s disease supported by PubMed identifiers at edges.

TP53INP2, which promotes the processing of APP by BACE1 and Gamma-secretase, emerges as another candidate (Fig. 7)^34^. Investigations have demonstrated that knockdown or antagonization of TP53INP2 reduces secretase activities and ameliorates Abeta pathology and Abeta-dependent behavioural deficits. Hence, intervention with TP53INP2 interaction can also serve as a new strategy against AD. Dextromethorphan (NMDA receptor agonist) is an established antitussive drug that targets TP53INP2 and a clinical trial (www.clinicalTrials.gov ID: NCT01584440) is already running to test if Dextromethorphan can be used as a drug for treating AD.

**Figure 8:**
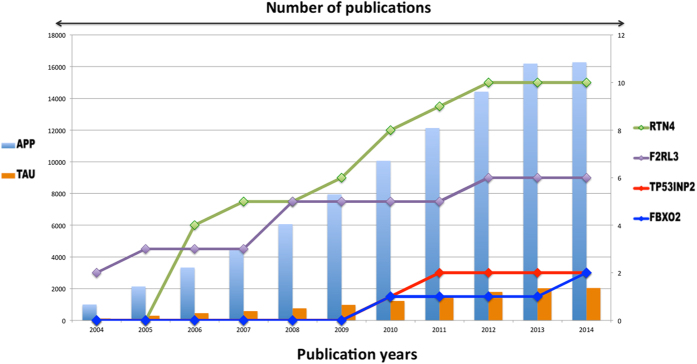
Number of publications in a chronological order-supporting role of established and emerging candidates in Alzheimer’s disease. Figure shows a schematic representation of the number of publications backing up the role of APP and tau (bar graph) in AD as compared to the emerging candidates (line graph) revealed in this study. Abbreviations mentioned stands for Amyloid beta (A4) precursor protein (APP), Microtubule associated protein tau (MAPT), Reticulon 4 (RTN4), F-box protein 2 (FBXO2), Thrombin Receptor-Like 3 (F2RL3), Tumor Protein P53 Inducible Nuclear Protein (TP53INP2).

All these proposed candidates look very interesting in a functional context and ongoing clinical trials certainly add up to the confidence. However, studies highlighting these candidates and their role in pathology of AD barely exist in the literature as compared to the frequently investigated APP and MAPT proteins (Fig. 8). This imbalance between the numbers of articles reflects the publication bias towards APP and MAPT in the disease context.

We show here that any evidence indicative of a potential role for a particular protein in the disease mechanism could be very valuable, particularly when these lines of evidence are aggregated and placed into the context of disease progression as a mechanistic, context-sensitive model.

## Discussion

The computational evaluation and scoring of protein–protein interactions certainly offer potential to ascertain compelling candidates mentioned in the literature. Protein interaction analysis based on reliability assessment may also uncover unique, unforeseen functional roles for not so-well-known proteins. Particularly, in complex diseases like AD, we should aim at integrating both established and emerging knowledge in integrative models. Linking hypotheses to the established knowledge or background theory can strengthen the ability of hypothesis-driven predictions. Especially, encoding relevant knowledge into causal relationship models further confers enhanced interpretation power that is well suited to back up existing hypotheses. Integrative analysis of extracted knowledge in the context of AD mechanism is a promising approach towards identification of candidates most likely involved in the complex aetiology of the disease. We are aware that we increase the level of speculation when we generate these “emerging functional context models”, but at the same time, we generate testable hypotheses through this modelling and mining approach, which are guided by neighbouring established knowledge.

From our evaluations, it was clear that low-scored coherent interactions lying next to high scoring neighbors (knowledge cliff) are of value and had a higher likelihood to impact disease etiology. Moreover, functional annotations for the selected cliff-interaction must be taken into account, which further helps to refine the candidate selection. The major reason accountable for the low scoring interactions could be their recent discovery or an expert bias towards established trends. Interesting, and particularly surprising candidates are RTN4 and TP53INP2 that interact with BACE1, and were predicted using our approach but their relevance to mechanism of AD has been barely studied in the literature, although their interactions are targeted in clinical trials for testing potential AD drugs.

We have tried to assign reliability scores based on selection of parameters that accumulate information (including negative evidence) and are most suitable for protein interactions. These parameters were further weighted to reflect the joint belief of domain experts. Thus, our score calculation goes much beyond just measuring the number of studies reporting an interaction by providing as a rationale to find candidates partially neglected in the literature, particularly when they have been shown to be upstream regulator of a well-established candidate. The criteria used in this work for calculation of scores are well suited for application to any other disease domain, which extends the scope of its applicability beyond the AD discipline. We believe that the method developed here can provide support to the emerging knowledge existing in other research realms. The knowledge-based models built in this way provide a first concrete framework to start with and to support arguments for prioritization of existing hypotheses. In view of the rapid pace of scientific articles, our approach proposes a strategy that helps in mining and prioritization of interesting candidates that could shift the direction of research towards new and rewarding avenues.

## Conclusion

This work demonstrates that reliability assessment can serve as a rationale for detection of emerging knowledge niches embedded in a defined functional context. Mining relevant knowledge zones (Emerging + Established) in a network context and further modelling their causal relationships confers enhanced interpretation power that is well suited for identification and prioritization of potential drug target or biomarker candidates at a mechanistic level. In the future, we intend to apply this strategy to different disease areas and conduct additional research to set up automated alerts for identification of novel knowledge cliffs.

## Additional Information

**How to cite this article**: Malhotra, A. *et al.* Exploring novel mechanistic insights in Alzheimer's disease by assessing reliability of protein interactions. *Sci. Rep.* **5**, 13634; doi: 10.1038/srep13634 (2015).

## Supplementary Material

Supplementary Information

Supplementary dataset 1

Supplementary dataset 2
