# Supplementary material for: Exploring novel mechanistic insights in Alzheimer’s disease by assessing reliability of protein interactions
Source: Sci Rep. 2015 Sep 8;5:13634. doi: 10.1038/srep13634 (PMC4562155; doi:10.1038/srep13634)
Supplement: Supplementary Information [file srep13634-s1.pdf]

Exploring novel mechanistic insights in Alzheimer's disease by assessing reliability of protein interactions

Ashutosh Malhotra[a,c], Erfan Younesi[a], Sudeep Sahadevan[b], Joerg Zimmermann[c] and Martin Hofmann-Apitius[a,c*]

[a]Department of Bioinformatics, Fraunhofer Institute for Algorithms and Scientific Computing (SCAI), Schloss Birlinghoven, 53754 Sankt Augustin, Germany.

[b]European Molecular Biology Laboratory (EMBL), Meyerhofstraße 1, 69117 Heidelberg, Germany.

[c]Rheinische Friedrich-Wilhelms-Universität Bonn, Bonn-Aachen International Center for Information Technology, 53113, Bonn, Germany.

Enclosure: This document contains 7 supplementary files mentioned in the manuscript as supporting evidences.

Supplementary file 1

Annotation guidelines for curation of protein-protein interaction data

The goal of this exercise is to extract sentences (serving as evidences) existing in literature, which represents relevant protein-protein interaction (PPi) information.

Results of experiments performed to verify a particular PPi are often conveyed in scientific publications. We aim to utilize this wealth of available pubic data and extract PPi based information specific to a particular disease domain leading to the generation of a disease specific protein interaction network.

A Machine learning approach is used for initial extraction of sentences existing in literature related to particular disease. Since we want to extract all possible information existing in literature so the automated machine learning approach

was optimized to generate maximum recall. Figure 1 shows the output sentences generated by following a machine learning approach.

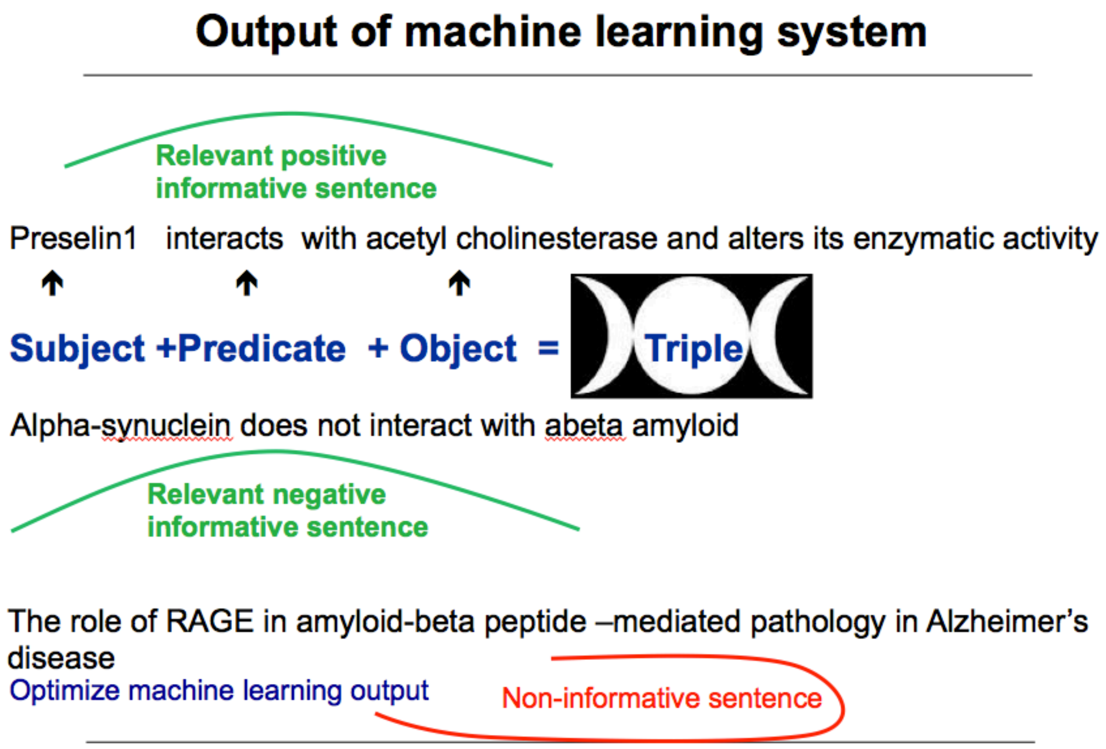

Figure1: Sentences extracted from biological literature using a Machine learning approach trained for relationship extraction.

Now as shown in Figure1, a automated work flow can also generate false positives. So, for the construction of a accurate disease specific interaction network "human curation" is necessary.

Task

You have been provided with sentences (output of a machine learning approach) which are believed to represent Protein-protein interaction(PPi) .

Protein–protein interactions occur when two or more proteins bind together, often to carry out their biological function.

Some examples:

"Within this complex, LGI1 binds selectively to a neuronal specific membrane protein, ADAM22 (a disintegrin and metalloprotease)".

"Presenilin-1 interacts with plakoglobin and enhances plakoglobin-Tcf-4 association."

Your task is to manually cross check the output of the automated approach (sentences) and see weather they really represent a protein- protein interaction or not (Specific to the disease assigned to you) and if they do represent (PPi) then further annotate them with the below mentioned concepts.

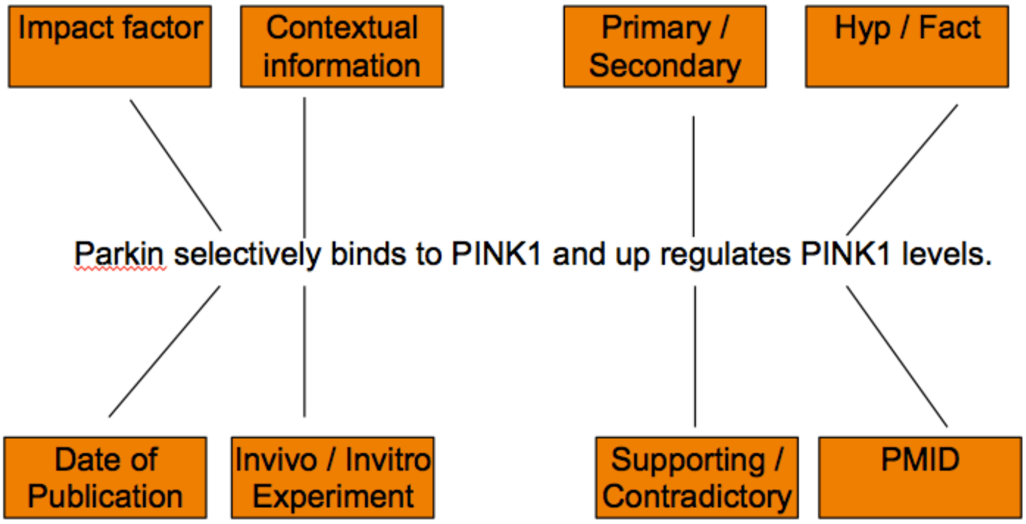

Figure 2: Various features for which each sentence representing valid PPI has been annotated

You can use a excel sheet with columns to do all your annotations with features like: contexual information, date of publication, Journal name, PMID, Impact factor of journal, Fact/Hypothesis, supporting/contradictory evidence, Invivo/invitro evidence.

Let go through each of these features in detail:

Task 1: Read the sentence carefully and determine weather this represent a PPi or not (relevance). If this represent PPi then assign a label 1 in front of it or if it does not represent PPi assign label 0 to it(If you assign a lable 0 to a sentence which you think does not contain PPi then you need not to do any further annotations for this sentence)

Note: Some time the sentence does not represent PPI but contains usefull information which we call as knowledge . Example:

P-glycoprotein, multi-drug resistance associated protein and major vault protein contribute to the pathogenesis of refractory epilepsy.

The sentence does not represent PPi but surely represent useful information about proteins that contribute to pathogenesis of refractory epilepsy.

All such sentences should be assigned a label 3 infront of them which means they represent knowledge.

Just for your information: Some times it may be difficult to classify what to consider knowledge /what not . In this case ask your self a question is this sentence helping me know some insights into the disease mechanism or can this information be useful for drug discovery purposes. Anything which is useful for us in getting new insight into the disease is knowledge for us.

Task 2: Determine weather information represented in the PPI really belongs to the disease assigned to you(Contextual information).

Suppose you are annotating a corpus of Alzheimer PPI's .Sometimes what happens in abstracts is that there is just mention of Alzheimer as a word in the abstract and the whole abstract is taking about normal brain phenomenon.

So you have to make sure that the sentence is definitely taken from abstract which is taking about alzheimer's disease.

Task 3: add PMID, Date of publication, Journal name and impact factor of the journal information to your annotation.

*Impact factor is one of the most controversial features included in the scoring, as there exist a mixed view among scientists to consider this as a parameter for judging quality. Although finally going on with the consensus and based on the arguments that this factor must be taken into account especially when judging reliability of literature derived information we have included it in our scoring.

Task 4: Classify weather the sentence is a fact or a hypothesis

Example: You are living in Bonn
Nature is beautiful
Angela merkel is a German
These are all facts

Hypothesis/Speculation:
Tomorrow I may go to Koln
I don't know if nature will also be beautiful in Sahara desert
Angela merkel might again become German chancellor

All these sentences are speculations because we are not sure if the are happening or not. Remember words like may, might, could be, suggest etc are used to represent speculation in text

So for each PPi sentence you have to check if it is a fact or speculation.

"GRK2 may hyperphosphorylates tau in tauopathies" (Hypothesis)

"Presenilin-1 interacts with plakoglobin and enhances plakoglobin-Tcf-4 association" (Fact)

Task 5: Check weather the interaction sentence mentioned is a supporting evidence or a contraction

If you are considering a interaction of A-B(Two proteins)

Then A interacts with B is supporting evidence

"In mouse, we found a high avidity binding of Abeta peptides to ACHE."

A does not interact with B is a contraction (opposite to supporting evidence)

"Our protein interaction experiment argues against interaction between APP and ACHE"

Task 6: Classify if a PPi is supported by a invivo evidence or In vitro evidence.
In this case you will have to read the full text to check weather the experiment conducted is invivo or invitro.

In Vivo experiment refers to a medical test, experiment or procedure that is done on a living organism, such as a laboratory animal or human.

In Vitro refers to experiments done within a glass or culture medium, observable in a test tube, in an artificial environment outside the living organism;

Or

Biological processes or reactions that would normally occur within an organism but here are made to occur in an artificial environment, i.e. A laboratory

You must also annotate the "interaction detection method" used to confirm a particular PPi existing in literature. Most cases particular PPI is backed up with multiple interaction detection methods. Please mention all of them with the interaction separated by comma along with their ID's as mentioned in PPIO ontology (http://bioportal.bioontology.org/ontologies/PPIO) to make sure you are annotating a relevant PPI detection method.

Supplementary file 2

Guidelines for ranking of various knowledge bins from an expert point of view

The aim of this survey is to rank various parameter combinations, which provides confidence to extracted 'Protein-protein interactions related knowledge' present in literature.

Using different parameter combinations (bins), we want to create a scoring function that provides a rational for confidence assessment of a protein-protein interaction reported in literature. Hence, leading to differentiation of established knowledge (supporting information and contradictions), emerging knowledge and novel predictions.

You have been provided below a list of 12 bins that you have to rank starting from maximum to minimum priority.

Each bin presented below represents a particular type of evidence supporting protein-protein interactions (PPI) and it is composed of the following entities:

- **Fact**: Sentence mentioned in literature is a fact e.g.
  - FGF-20 selectively activates tyrosine hydroxylase in calbindin-negative neurons.
- **Hypo**: Sentence mentioned in literature is a speculation or hypothesis e.g.
  - FGF-20 selectively might activate tyrosine hydroxylase in calbindin-negative neurons.
- **Invivo:** The experiment showing PPI was conducted in human or animal (mouse or rat) as model organism.
- **Human:** Experiment performed in humans
- **Animal:** Experiment performed using mouse or rat as a model organism.
- **Invitro:** Experiment performed in a controlled environment outside of a living organism. (http://mmbr.asm.org/content/59/1/94.full.pdf)
  - **Physical methods:** Physical methods used for Protein protein interaction includes:
    - Protein affinity chromatography
    - Affinity bloating
    - Immunoprecipitation
    - Cross linking
  - **Library based methods:** Library based methods used for Protein protein interaction includes:
    - Protein probing
    - Phage display
    - Two hybrid system
  - **Genetic methods:** Genetic methods used for Protein protein interaction includes:
    - Extragenic suppressors
    - Synthetic lethal effect

- Over production phenotypes

  - **Others :** All other methods (in-silico etc) apart from one listed above will be mapped to this class.

Note: Although, the techniques mentioned under invitro section (Physical, library based and genetic methods) are all done in laboratory but you are required to rank all of them based on the amount of trust you have in them.

A 'Bin' is composed of different combinations of above exemplified entities represent the following:

**Fact|Invivo|Human:** A evidence or statement extracted from given article is a fact supported by the study mentioned within the same article and the model organism for this Invivo experiment is human
**Fact|Invivo|Animal:** A evidence or statement extracted from given article is a fact supported by the study mentioned within the same article and the model organism used for this Invivo experiment is animal(Mouse/Rat)
**Hypo|Invivo|Human:** A evidence or statement extracted from given article is a Hypothesis supported by the study mentioned within the same article and the model organism is human
**Hypo|Invivo|Animal:** A evidence or statement extracted from given article is a hypothesis supported by the study mentioned within the same article and the model organism for this Invivo experiment is animal(Mouse/Rat)
**Fact|Invitro|Physicochemical methods:** A evidence or statement extracted from given article is a fact supported by the study mentioned within the same article and the study mentioned used a Physical techniques for PPI detection
**Hypo|Invitro|Physicochemical methods:** A evidence or statement extracted from given article is a Hypothesis supported by the study mentioned within the same article and the study mentioned used a Physical techniques for PPI detection
**Fact|Invitro|Library based methods:** A evidence or statement extracted from given article is a fact supported by the study mentioned within the same article and the study mentioned used Library based methods for PPI detection
**Hypo|Invitro|Library based methods:** A evidence or statement extracted from given article is a Hypothesis supported by the study mentioned within the same article and the study mentioned used Library based methods for PPI detection
**Fact|Invitro|Genetic methods:** A evidence or statement extracted from given article is a fact supported by the study mentioned within the same article and the study mentioned used Genetic methods for PPI detection
**Hypo|Invitro|Genetic methods:** A evidence or statement extracted from given article is a hypothesis supported by the study mentioned within the same article and the study mentioned used Genetic methods for PPI detection
**Fact|Invitro|other:** A evidence or statement extracted from given article is a fact supported by the study mentioned within the same article and the study mentioned used other methods (in-silico or something else) apart from one mentioned above for PPI detection
**Hypo|Invitro|other:** A evidence or statement extracted from given article is a fact supported by the study mentioned within the same article and the study mentioned used other methods (in-silico or something else) apart from one mentioned above for PPI detection

You are requested to rank all of these bins (representing confidence of statements derived from literature with evidences) based on your expert opinion. Hence considering the above-mentioned details please assign suitable rank to each bin. To show you how you can rank these parameters based on your expertise, please see the below table that has been assigned ranks to demonstrate an example:

S.No	Bin	Rank
**1**	**Fact|Invivo|Human**	1
**2**	**Fact|Invivo|Animal**	2
3	**Hypo|Invivo|Human**	3
4	**Hypo|Invivo|Animal**	5
5	**Fact|Invitro|Physical methods**	4
6	**Hypo|Invitro|Physical methods**	8
7	**Fact|Invitro|Library based methods**	7
8	**Hypo|Invitro|Library based methods**	10
9	**Fact|Invitro|Genetic methods**	6

10	**Hypo|Invitro|Genetic methods**	9
11	**Fact|Invitro|other**	11
12	**Hypo|Invitro|other**	12

Please assign a rank to the bin as described above based on your experience and priority:

**S.No**	**Bin**	**Rank**
**1**	**Fact|Invivo|Human**	
**2**	**Fact|Invivo|Animal**	
3	**Hypo|Invivo|Human**	
4	**Hypo|Invivo|Animal**	
5	**Fact|Invitro|Physical methods**	
6	**Hypo|Invitro|Physical methods**	
7	**Fact|Invitro|Library based methods**	
8	**Hypo|Invitro|Library based methods**	
9	**Fact|Invitro|Genetic methods**	
10	**Hypo|Invitro|Genetic methods**	
11	**Fact|Invitro|other**	
12	**Hypo|Invitro|other**	

Thanks for your help and all the best !

<u>Supplementary file 3</u>

Protein interaction network specific to Alzheimer's disease, which consists of 301 nodes (proteins) and 339 edges (protein interactions). Each edge present in the network is directed and has been assigned with a reliability score.

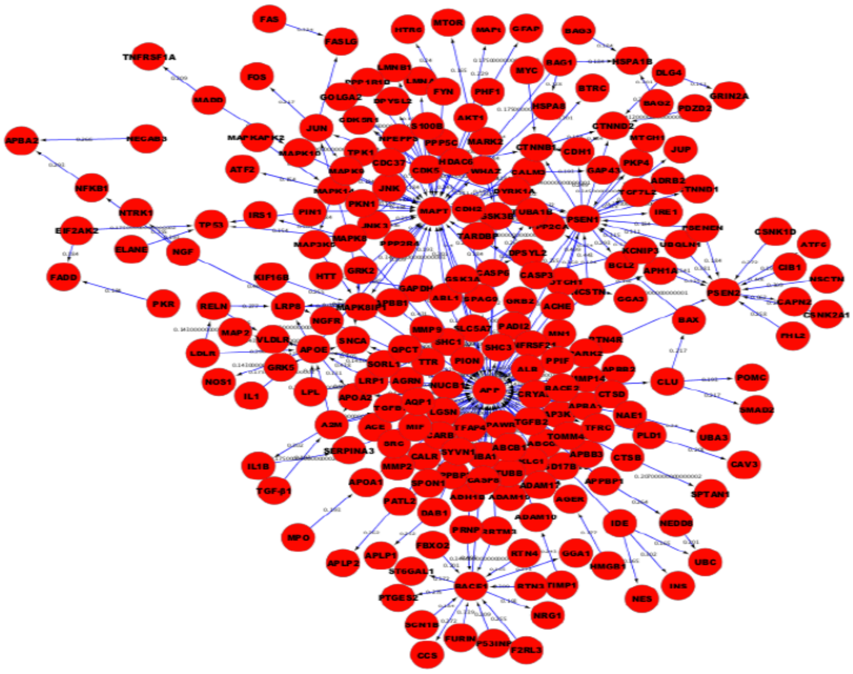

Protein1	Protein2	Directionality	Relevence	Pubmed.id	Date	Journal.Name	Impact.Factor	Fact	Invivo.Hum	Invivo.Anim	Invitro	Sentence	norm_score
BACE1	APP	BACE1-APP	1	10500121	1999	Proc Natl Acad Sci U S	9.681	fact			Co-immuno	A pathogenic mutation at codons 670/671 in APP (APP Swedish) lea	1
APOE	APP	APOE-APP	1	21297948	2011	PLoS One	4.01	fact	FRET		Co-immuno	APOE interacts with APP in human Alzheimers brain	0.84
GSK3B	MAPT	GSK3B-MAP'	1	16257959	2005	Journal of Biological C	4.773	hypo		Co-immuno	Pull down a	These results, respectively, indicated that GSK-3beta is responsible	0.626
PSEN1	APP	PSEN1-APP	1	10653282	1999	Eur Arch Psychiatry Cl	3.494	fact			Solid phase	Instead, membrane associated carboxyterminal fragments generate	0.586

Figure 3: Protein interaction network specific to Alzheimer's disease.

The network is available for download at the following URL:
http://www.scai.fraunhofer.de/de/geschaeftsfelder/bioinformatik/downloads.html

Network file available in (.xgmml format) can be opened and visualized using Cytoscape_Version2.8.3 onwards.

To open the file with the Cytoscape; please follow these steps:

1. Download and install the appropriate Cytoscape version
2. Open Cytoscape
3. Please go to the file menu and select import Network(multiple file types) option.
4. Please browse through the downloaded network file.
5. Then select the network file and you will see the network in your cytoscape window.

Supplementary file 4

The random network constructed from test corpus annotation is available for download at the following URL:
http://www.scai.fraunhofer.de/de/geschaeftsfelder/bioinformatik/downloads.html

Network file available in (.xgmml format) can be opened and visualized using Cytoscape_Version2.8.3 onwards.

To open the file with the Cytoscape; please follow these steps:

1. Download and install the appropriate Cytoscape version
2. Open Cytoscape
3. Please go to the file menu and select import Network(multiple file types) option.
4. Please browse through the downloaded network file.
5. Then select the network file and you will see the network in your cytoscape window.

Supplementary file 5

Evidences extracted from literature using biomarker terminology where particular candidates have been mentioned as Biomarkers for Alzheimer's disease.

CASP6

- increased expression of CASP6 in frontal cortex correlates with increased apoptosis associated with Alzheimer disease [PMID: 15277226]
- increased expression of CASP6 in frontal cortex correlates with nerve degeneration associated with Alzheimer disease [PMID: 15277226]
- increased expression of CASP6 in temporal cortex correlates with increased apoptosis associated with Alzheimer disease [PMID: 15277226]
- increased expression of CASP6 in temporal cortex correlates with nerve degeneration associated with Alzheimer disease [PMID: 15277226]
- mislocalization of CASP6 protein correlates with nerve degeneration associated with Alzheimer disease [PMID: 15277226]

TNFRSF21

- increased expression of TNF in blood correlates with Alzheimer disease [PMID: 14975597]
- increased expression of TNF in cerebrospinal fluid correlates with Alzheimer disease [PMID: 15936505]
- increased expression of TNF in cerebrospinal fluid correlates with early stage or low grade form of Alzheimer disease [PMID: 11164279]
- increased expression of TNF in microglia may correlate with inflammation associated with Alzheimer disease [PMID: 11424194 , 12404514]
- increased expression of TNF in serum correlates with increased T cell activation associated with Alzheimer disease [PMID: 12493553]
- polymorphism in the TNF gene correlates with increased occurrence of disease susceptibility associated with Alzheimer disease [PMID: 11121190]
- polymorphism in the TNF gene correlates with late onset form of Alzheimer disease [PMID: 11378846]

SERPINA3

- increased expression of SERPINA3 in brain correlates with Alzheimer disease [PMID: 9688331]
- increased expression of SERPINA3 in brain correlates with amyloidosis associated with Alzheimer disease [PMID: 1702930]
- increased expression of SERPINA3 in cerebrospinal fluid correlates with Alzheimer disease [PMID: 17761554]
- increased expression of SERPINA3 in plasma correlates with increased severity of cognition disorders associated with Alzheimer disease [PMID: 12509851]
- increased expression of SERPINA3 in serum correlates with Alzheimer disease [PMID: 1387300]
- increased expression of SERPINA3 in serum correlates with dementia associated with Alzheimer disease [PMID: 8157733]
- polymorphism in the SERPINA3 gene correlates with decreased occurrence of disease susceptibility associated with Alzheimer disease [PMID: 11941486]

CCL2

- increased expression of CCL2 in astrocytes may correlate with early stage or low grade form of Alzheimer disease [PMID: 11424194]
- increased expression of CCL2 in astrocytes may correlate with inflammation associated with Alzheimer disease [PMID: 11424194]
- increased expression of CCL2 in brain correlates with inflammation associated with Alzheimer disease [PMID: 11754990]

GRIN2A

- decreased expression of GRIN2A in frontal cortex correlates with Alzheimer disease [PMID: 11038245]
- decreased expression of GRIN2A in hippocampus correlates with Alzheimer disease [PMID: 12127670]

RELN

- increased expression of RELN in frontal cortex correlates with Alzheimer disease [PMID: 16567613]
- increased expression of RELN protein correlates with Alzheimer disease [PMID: 12645087]

CTSB

- Our data indicate that CGA-activated microglia can trigger neuronal apoptosis and that this may be mediated through the secretion of cathepsin B. [PMID:11238732]
- Our results suggest that cathepsin B plays novel roles in the metabolism of APP and that an inhibition of APP phosphorylation is an attractive therapeutic target for Alzheimer's disease. [PMID:21746863]
- We hypothesize that cathepsin B levels can discriminate persons with AD or MCI from healthy controls.[PMID:20930303]

SORL1

- decreased expression of SORL1 in brain correlates with increased neuron apoptosis associated with Alzheimer disease [PMID: 16452683]
- decreased expression of SORL1 in brain correlates with increased severity of amyloidosis associated with Alzheimer disease [PMID: 17589324]
- polymorphism in the SORL1 gene correlates with increased occurrence of late onset form of Alzheimer disease [PMID: 17220890]

TGFB1

- increased expression of TGFB1 in brain correlates with cerebral amyloid angiopathy associated with Alzheimer disease [PMID: 9335500]
- increased expression of TGFB1 in cerebrospinal fluid may correlate with abnormal beta-amyloid metabolic process associated with Alzheimer disease [PMID: 15331151]
- polymorphism in the TGFB1 gene may correlate with late onset form of Alzheimer disease [PMID: 10914688]

BAX

- decreased expression of BAX in hippocampus correlates with increased neurons survival associated with Alzheimer disease [PMID: 9098548]

IL1B

- increased expression of IL1B in blood correlates with Alzheimer disease [PMID: 14975597]

- increased expression of IL1B in brain correlates with Alzheimer disease [PMID: 15176485]
- increased expression of IL1B in cerebrospinal fluid correlates with increased incidence of non-familial form of Alzheimer disease [PMID: 8787820]
- increased expression of IL1B in cerebrospinal fluid may correlate with Alzheimer disease [PMID: 7693756]
- increased expression of IL1B in hippocampus correlates with Alzheimer disease [PMID: 7838374]
- single nucleotide polymorphism in the IL1B gene correlates with disease progression associated with Alzheimer disease [PMID: 15212826]
- single nucleotide polymorphism in the IL1B gene may correlate with genetic predisposition to disease associated with Alzheimer disease [PMID: 15653174]

GSK3b

- increased expression of GSK3B in brain correlates with Alzheimer disease [PMID: 10486203]
- increased expression of GSK3B in leukocytes correlates with Alzheimer disease [PMID: 15555766]
- increased expression of GSK3B in neurons may correlate with increased protein amino acid phosphorylation associated with Alzheimer disease [PMID: 8930358]
- increased phosphorylation of GSK3B correlates with Alzheimer disease [PMID: 15126504]

TUBA1B

- The alpha-tubulin mRNA was strongly expressed in neurons in the gyrus dentatus,[PMID: 7479369]

PTGES2

- increased expression of PTGS2 in hippocampus correlates with Alzheimer disease [PMID: 12770691]
- increased expression of PTGS2 in neurons correlates with Alzheimer disease [PMID: 12770691]
- increased expression of PTGS2 in pyramidal cells correlates with Alzheimer disease [PMID: 10412020]

MAPT

- abnormal expression of MAPT epitope correlates with disease progression associated with Alzheimer disease [PMID: 15356202]
- abnormal expression of MAPT epitope correlates with neurofibrillary tangles associated with Alzheimer disease [PMID: 15356202]
- abnormal mRNA splicing of MAPT correlates with non-familial form of Alzheimer disease [PMID: 16371011]
- abnormal phosphorylation of MAPT correlates with Alzheimer disease [PMID: 1826835]
- alternative form of MAPT mRNA correlates with disease progression associated with Alzheimer disease [PMID: 16478530]
- increased expression of MAPT in cerebrospinal fluid correlates with disease progression associated with Alzheimer disease [PMID: 16843497]
- mislocalization of MAPT protein correlates with Alzheimer disease [PMID: 1707726]
- nitration of MAPT may correlate with Alzheimer disease [PMID: 17050703]
- polymorphism in the MAPT gene correlates with early onset form of Alzheimer disease [PMID: 15106853]
- polymorphism in the MAPT locus correlates with disease susceptibility associated with Alzheimer disease [PMID: 16000317]
- single nucleotide polymorphism in the MAPT locus may correlate with Alzheimer disease [PMID: 17179995]

IL6

- decreased secretion of IL6 correlates with increased severity of dementia associated with Alzheimer disease [PMID: 12928049]
- increased expression of IL6 in blood correlates with Alzheimer disease [PMID: 12928051]
- increased expression of IL6 in brain correlates with inflammation associated with Alzheimer disease [PMID: 11754990]
- increased expression of IL6 in cerebrospinal fluid correlates with dementia associated with Alzheimer disease [PMID: 10865077]
- increased expression of IL6 in cerebrospinal fluid correlates with increased acute-phase response associated with Alzheimer disease [PMID: 8787820]
- increased expression of IL6 in cerebrospinal fluid correlates with non-familial form of Alzheimer disease [PMID: 8787820]
- increased expression of IL6 in microglia may correlate with Alzheimer disease [PMID: 11424194]
- increased expression of IL6 in plasma correlates with increased incidence of disease susceptibility associated with Alzheimer disease [PMID: 11992567]
- increased expression of IL6 in plasma may correlate with inflammation associated with Alzheimer disease [PMID: 10674995]
- increased secretion of IL6 correlates with inflammation associated with Alzheimer disease [PMID: 11754990]

- polymorphism in the IL6 gene correlates with decreased occurrence of non-familial form of Alzheimer disease [PMID: 10319892]

IL10

- increased expression of IL10 in microglia may correlate with Alzheimer disease [PMID: 12404514]
- polymorphism in the IL10 promoter correlates with increased incidence of non-familial form of Alzheimer disease [PMID: 14746878]
- single nucleotide polymorphism in the IL10 promoter correlates with Alzheimer disease [PMID: 15212825]

A2M

- increased expression of A2M in brain correlates with Alzheimer disease [PMID: 1712317]
- increased expression of A2M in plasma correlates with more severe form of Alzheimer disease [PMID: 17071923]
- increased expression of A2M protein may correlate with increased cell death associated with Alzheimer disease [PMID: 10072300]
- polymorphism in the A2M gene correlates with increased incidence of disease susceptibility associated with Alzheimer disease [PMID: 15931081]

NTRK1

- decreased expression of NTRK1 in caudate nucleus correlates with Alzheimer disease [PMID: 10993689]
- decreased expression of NTRK1 in frontal cortex correlates with Alzheimer disease [PMID: 9225742]
- decreased expression of NTRK1 in neurons correlates with Alzheimer disease [PMID: 9051746]
- decreased expression of NTRK1 in parietal cortex correlates with Alzheimer disease [PMID: 9507943]
- decreased expression of NTRK1 in telencephalon correlates with Alzheimer disease [PMID: 9225742]
- Decreased TrkA gene expression in cholinergic neurons of the striatum and basal forebrain of patients with Alzheimer's disease [PMID:9184126]

NGF

- increased expression of NGF in frontal cortex correlates with Alzheimer disease [PMID: 8501520]
- increased expression of NGF in occipital cortex correlates with Alzheimer disease [PMID: 8501520]

PSEN1

- decreased expression of PSEN1 in hippocampus correlates with Alzheimer disease [PMID: 9067452]
- decreased protein binding of PSEN1 correlates with Alzheimer disease [PMID: 10754226]
- deletion mutation in the PSEN1 promoter correlates with increased occurrence of early onset form of Alzheimer disease [PMID: 10655540]
- increased expression of PSEN1 in brain correlates with Alzheimer disease [PMID: 17586478]
- increased expression of PSEN1 mRNA correlates with Alzheimer disease [PMID: 18256261]
- increased expression of PSEN1 protein correlates with cerebral amyloid angiopathy associated with Alzheimer disease [PMID: 9573389]
- mutation in the PSEN1 gene correlates with familial form of Alzheimer disease [PMID: 15776278]
- mutation in the PSEN1 gene correlates with increased severity of neurofibrillary tangles associated with Alzheimer disease [PMID: 15946688]
- mutation in the PSEN1 gene may correlate with abnormal protein amino acid dephosphorylation associated with Alzheimer disease [PMID: 8805118]
- polymorphism in the PSEN1 locus correlates with increased occurrence of late onset form of Alzheimer disease [PMID: 8596269]
- single nucleotide polymorphism in the PSEN1 gene correlates with disease susceptibility associated with Alzheimer disease [PMID: 16938285]

ACHE

- abnormal expression of ACHE in cerebrospinal fluid correlates with Alzheimer disease [PMID: 9351648]
- abnormal glycosylation of ACHE correlates with Alzheimer disease [PMID: 10098867]
- decreased acetylcholinesterase activity of ACHE correlates with Alzheimer disease [PMID: 15854764]
- decreased acetylcholinesterase activity of ACHE correlates with sleep-wake transition disorders associated with Alzheimer disease [PMID: 17539952]
- decreased expression of ACHE in brain correlates with Alzheimer disease [PMID: 9269216 , 10939570]

- decreased expression of ACHE in brain correlates with late onset form of Alzheimer disease [PMID: 10939570]
- decreased expression of ACHE in cerebral cortex correlates with Alzheimer disease [PMID: 7830069]
- decreased expression of ACHE in lymphocytes correlates with non-familial form of Alzheimer disease [PMID: 8195795]
- decreased expression of ACHE in neurons correlates with Alzheimer disease [PMID: 1508295]
- decreased expression of ACHE in pyramidal cells correlates with nerve degeneration associated with Alzheimer disease [PMID: 1508295]
- decreased plasma membrane localization of ACHE correlates with Alzheimer disease [PMID: 1491735]
- increased expression of ACHE in cerebrospinal fluid correlates with dementia associated with Alzheimer disease [PMID: 1671469]
- increased expression of ACHE in frontal cortex correlates with Alzheimer disease [PMID: 9681463]

SNCA

- increased expression of SNCA in brain correlates with Alzheimer disease [PMID: 8546207]
- increased expression of SNCA in frontal cortex correlates with early stage or low grade form of Alzheimer disease [PMID: 8782917]
- increased expression of SNCA in neurons correlates with neurofibrillary tangles associated with Alzheimer disease [PMID: 10727692]
- increased expression of SNCA in neurons correlates with tauopathies associated with Alzheimer disease [PMID: 10727692]
- increased inclusion body localization of SNCA correlates with Lewy body disease associated with Alzheimer disease [PMID: 11117482]
- increased nitration of SNCA correlates with Lewy body disease associated with Alzheimer disease [PMID: 11062131]

LRP8

- polymorphism in the LRP8 gene may correlate with Alzheimer disease [PMID: 12399018]
- We speculated that another member of this LDL receptor family, LRP8 (also called apolipoprotein E receptor 2 or ApoER2), which is predominantly expressed in brain, might be associated with Alzheimer's disease. [PMID: 12399018]
- We also found that expression of LRP8 increased APP association with lipid rafts and increased gamma-secretase activity, both of which might contribute to increased Abeta production. [PMID: 17620134]

PIN1

- abnormal expression of PIN1 protein correlates with early stage or low grade form of Alzheimer disease [PMID: 14572447]
- abnormal tau protein binding of PIN1 correlates with Alzheimer disease [PMID: 10391244]
- increased oxidation of PIN1 correlates with disease progression associated with Alzheimer disease [PMID: 16466929]
- mislocalization of PIN1 protein correlates with Alzheimer disease [PMID: 10391244]

F2RL3

- abnormal expression of F2 protein may correlate with nerve degeneration associated with Alzheimer disease [PMID: 8544905]
- mislocalization of F2 protein correlates with Alzheimer disease [PMID: 1491781]

IL2

- increased expression of IL2 in hippocampus correlates with Alzheimer disease [PMID: 7838374]
- increased secretion of IL2 correlates with more severe form of Alzheimer disease [PMID: 8034754]

CTNND2

- abnormal protein binding of CTNND2 may correlate with early onset form of Alzheimer disease [PMID: 11447843]
- CTNND2
  based adhesion enhances Abeta release and decreases Abeta42/40 ratio [PMID: 19046403]

ACE

- increased expression of ACE in parietal cortex correlates with Alzheimer disease [PMID: 11445253]
- increased expression of ACE in temporal cortex correlates with Alzheimer disease [PMID: 1664329]
- polymorphism in the ACE gene correlates with Alzheimer disease [PMID: 10643899]

PRNP

- polymorphism in the PRNP gene correlates with increased occurrence of disease susceptibility associated with Alzheimer disease [PMID: 12601712]
- polymorphism in the PRNP gene correlates with increased occurrence of early onset form of Alzheimer disease [PMID: 15277640]

POMC

- abnormal secretion of POMC correlates with Alzheimer disease [PMID: 1846870]
- alternative form of POMC protein correlates with Alzheimer disease [PMID: 2974929]
- decreased expression of POMC in cerebrospinal fluid correlates with dementia associated with Alzheimer disease [PMID: 2840605]

BACE1

- increased expression of BACE1 protein correlates with Alzheimer disease [PMID: 12112088]
- polymorphism in the BACE1 gene correlates with increased occurrence of disease susceptibility associated with Alzheimer disease [PMID: 15931081]

DLG4

- decreased expression of DLG4 in brain may correlate with Alzheimer disease [PMID: 15979210]
- decreased synapse localization of DLG4 correlates with Alzheimer disease [PMID: 15509549]

NGFR

- decreased expression of NGFR in hippocampus correlates with increased occurrence of early onset form of Alzheimer disease [PMID: 1436650]
- decreased expression of NGFR in neurons correlates with Alzheimer disease [PMID: 10683291]
- decreased expression of NGFR in telencephalon correlates with Alzheimer disease [PMID: 10683291]
- increased expression of NGFR in cerebral cortex correlates with Alzheimer disease [PMID: 1309947]

CLU

- alternative form of CLU protein correlates with Alzheimer disease [PMID: 14559363]
- decreased glycosylation of CLU correlates with increased beta-amyloid formation associated with Alzheimer disease [PMID: 16490286]
- increased expression of CLU in cerebrospinal fluid correlates with increased microtubule cytoskeleton organization associated with Alzheimer disease [PMID: 16490286]
- increased expression of CLU in frontal cortex correlates with Alzheimer disease [PMID: 9878186]
- increased expression of CLU in hippocampus correlates with Alzheimer disease [PMID: 1702645]

CDK5

- increased expression of CDK5 in neurons correlates with Alzheimer disease [PMID: 10620662]
- increased expression of CDK5 in neurons correlates with early stage or low grade form of Alzheimer disease [PMID: 9666145]
- single nucleotide polymorphism in the CDK5 gene correlates with disease susceptibility associated with Alzheimer disease [PMID: 15917097]

TP53

- abnormal expression of TP53 in fibroblasts correlates with defective response to hydrogen peroxide associated with Alzheimer disease [PMID: 12118068]
- abnormal expression of TP53 in fibroblasts correlates with non-familial form of Alzheimer disease [PMID: 12118068]
- increased expression of TP53 in brain correlates with increased DNA fragmentation during apoptosis associated with Alzheimer disease [PMID: 9395128]

GRK5

- Recent studies have indicated the possible involvement of GRK, primarily altered GRK2 and GRK5, dysfunction in the pathogenesis of AD. [20730384]
- Altogether, these findings indicate that GRK5 deficiency accelerates β-amyloidogenic APP processing and Aβ accumulation in APPsw mice via impaired cholinergic activity and that presynaptic M2 hyperactivity is the specific target for eliminating the pathologic impact of GRK5 deficiency. [21041302]

- GRK5 alteration may further increase beta amyloid production in Alzheimer's disease and exaggerates brain inflammation, possibly even the basal forebrain cholinergenic degeneration [20730384]

PSEN2

- alternative form of PSEN2 mRNA correlates with abnormal pyramidal cells structure associated with Alzheimer disease [PMID: 12133587]
- decreased expression of PSEN2 in forebrain correlates with Alzheimer disease [PMID: 8918895]
- decreased expression of PSEN2 in hippocampus correlates with early stage or low grade form of Alzheimer disease [PMID: 10891593]
- increased expression of PSEN2 in glial cells correlates with Alzheimer disease [PMID: 9067452]
- missense mutation in the PSEN2 gene correlates with idiopathic form of Alzheimer disease [PMID: 9384602]

APOE

- abnormal beta-amyloid metabolic process associated with APOE correlates with increased occurrence of genetic predisposition to disease associated with Alzheimer disease [PMID: 10939571]
- decreased expression of APOE in cerebrospinal fluid correlates with Alzheimer disease [PMID: 10823584]
- increased expression of APOE in plasma correlates with Alzheimer disease [PMID: 10208564]
- increased expression of APOE mRNA correlates with Alzheimer disease [PMID: 17586478]
- increased expression of APOE protein may correlate with decreased neurons function associated with Alzheimer disease [PMID: 10694577]
- polymorphism in the APOE gene correlates with decreased response to oxidative stress associated with Alzheimer disease [PMID: 10671320]
- polymorphism in the APOE gene correlates with early onset form of Alzheimer disease [PMID: 17485647]
- polymorphism in the APOE gene correlates with increased occurrence of delusions associated with Alzheimer disease [PMID: 16841077]
- polymorphism in the APOE gene correlates with increased occurrence of depression associated with Alzheimer disease [PMID: 17337010]
- polymorphism in the APOE gene correlates with increased occurrence of early onset form of Alzheimer disease [PMID: 8786847 , 11311499]
- polymorphism in the APOE gene correlates with increased occurrence of genetic predisposition to disease associated with Alzheimer disease [PMID: 17280645 , 17498878 , 17553421]
- polymorphism in the APOE gene correlates with increased occurrence of irritable mood associated with Alzheimer disease [PMID: 16841077]

- polymorphism in the APOE promoter correlates with increased occurrence of genetic predisposition to disease associated with Alzheimer disease [PMID: 12105308]
- single nucleotide polymorphism in the APOE enhancer correlates with increased occurrence of cognition disorders associated with Alzheimer disease [PMID: 17613540]
- single nucleotide polymorphism in the APOE gene correlates with late onset form of Alzheimer disease [PMID: 17317784]

TTR

- abnormal expression of TTR in cerebrospinal fluid correlates with Alzheimer disease [PMID: 11973456]
- decreased expression of TTR in cerebrospinal fluid correlates with Alzheimer disease [PMID: 9559653]

LDLR

- Our findings suggest that LDLR gene may be associated with AD risk and is a CSF biomarkers, especially in women.[PMID:17239995]
- increasing glial LDLR levels may promote Aβ degradation within the brain.[22383525]
- Therefore, these results identify LDLR as a receptor that mediates Aβ uptake and clearance by astrocytes, and provide evidence that increasing glial LDLR levels may promote Aβ degradation within the brain. [PMID: 22383525]
- These findings suggest that increasing LDLR levels may represent a novel AD treatment strategy.[PMID: 20005821]
- We found that over-expression of APP may cause increase in both LDLR mRNA and protein levels in APP transfected H4 neuroglioma cells compared to H4 controls.[20049331]

GRM1

- decreased expression of GRM1 in brain may correlate with dementia associated with Alzheimer disease [PMID: 15949941]
- These data suggest that group II mGluR may trigger synaptic activation of all three secretases and that suppression of group II mGluR signaling may be a therapeutic strategy for selectively reducing synaptic generation of Abeta(42). [PMID:20237257]
- induced stimulation of GRM1 protein may correlate with abnormal signal transduction associated with Alzheimer disease [PMID: 12054503]

APP

- abnormal expression of APP in cerebrospinal fluid correlates with non-familial form of Alzheimer disease [PMID: 17049739]

- abnormal processing of APP correlates with cognition disorders associated with Alzheimer disease [PMID: 17356877]
- abnormal processing of APP correlates with familial form of Alzheimer disease [PMID: 16752394]
- abnormal processing of APP correlates with non-familial form of Alzheimer disease [PMID: 17049739]
- alternative form of APP protein correlates with decreased neurons survival associated with Alzheimer disease [PMID: 8752124]
- alternative form of APP protein correlates with dementia associated with Alzheimer disease [PMID: 1642473]
- alternative form of APP protein may correlate with decreased neurons survival associated with Alzheimer disease [PMID: 8752124]
- APP map position correlates with late onset form of Alzheimer disease [PMID: 11500807]
- decreased expression of APP in cerebrospinal fluid correlates with Alzheimer disease [PMID: 11552007 , 12486489]
- decreased expression of APP in plasma correlates with non-familial form of Alzheimer disease [PMID: 8347821]
- decreased expression of APP mRNA correlates with Alzheimer disease [PMID: 8294927]
- decreased extracellular region localization of APP correlates with abnormal protein processing associated with Alzheimer disease [PMID: 7957195]
- decreased secretion of APP correlates with abnormal amyloid precursor protein metabolic process associated with Alzheimer disease [PMID: 9872930]
- deletion mutation in the APP gene correlates with non-familial form of Alzheimer disease [PMID: 11238715]
- hypomethylation of the APP gene correlates with Alzheimer disease [PMID: 8746452]
- increased endosome localization of APP correlates with non-familial form of Alzheimer disease [PMID: 15465622]
- increased expression of APP in brain correlates with Alzheimer disease [PMID: 3159021]
- increased expression of APP in cerebrospinal fluid correlates with late onset form of Alzheimer disease [PMID: 17366635]
- increased expression of APP in frontal cortex correlates with nerve degeneration associated with Alzheimer disease [PMID: 1331685]
- increased expression of APP in lens correlates with increased occurrence of cataract associated with Alzheimer disease [PMID: 12699953]
- increased expression of APP in monocytes correlates with abnormal protein processing associated with Alzheimer disease [PMID: 10588572]
- increased expression of APP in plasma correlates with increased occurrence of brain infarction associated with Alzheimer disease [PMID: 16401840]
- increased expression of APP in temporal cortex correlates with Alzheimer disease [PMID: 17586478]
- increased expression of APP mutant protein correlates with autosomal dominant form of Alzheimer disease [PMID: 16432153]

- increased expression of APP mutant protein correlates with early onset form of Alzheimer disease [PMID: 12787077]
- increased expression of APP mutant protein correlates with familial form of Alzheimer disease [PMID: 16432153]
- increased extracellular region localization of APP may correlate with non-familial form of Alzheimer disease [PMID: 11238715]
- increased oxidation of APP correlates with Alzheimer disease [PMID: 16816122]
- increased presence of APP autoimmune antibody correlates with Alzheimer disease [PMID: 15212827]
- increased presence of APP autoimmune antibody correlates with dementia associated with Alzheimer disease [PMID: 17477976]
- increased protein binding of APP correlates with familial form of Alzheimer disease [PMID: 12054732]
- increased proteolysis of APP correlates with abnormal protein processing associated with Alzheimer disease [PMID: 7745619]
- increased proteolysis of APP correlates with Alzheimer disease [PMID: 8025536]
- increased proteolysis of APP correlates with familial form of Alzheimer disease [PMID: 7523115]
- missense mutation in the APP gene correlates with abnormal cytoskeleton organization associated with Alzheimer disease [PMID: 1465214]
- missense mutation in the APP gene correlates with abnormal protein processing associated with Alzheimer disease [PMID: 11528419]
- missense mutation in the APP gene correlates with autosomal dominant form of Alzheimer disease [PMID: 12552037]
- missense mutation in the APP gene correlates with decreased Purkinje cells survival associated with Alzheimer disease [PMID: 9171327]
- missense mutation in the APP gene correlates with early onset form of Alzheimer disease [PMID: 9328472 , 12552037]
- missense mutation in the APP gene correlates with increased incidence of early onset form of Alzheimer disease [PMID: 8247223]
- missense mutation in the APP gene correlates with increased protein secretion associated with Alzheimer disease [PMID: 7957938]
- missense mutation in the APP gene correlates with stroke associated with Alzheimer disease [PMID: 12163376]
- mutation in the APP gene correlates with increased severity of neurofibrillary tangles associated with Alzheimer disease [PMID: 15946688]
- mutation in the APP gene may correlate with abnormal protein processing associated with Alzheimer disease [PMID: 12395079]
- mutation in the APP promoter correlates with Alzheimer disease [PMID: 16685645]
- polymorphism in the APP gene correlates with early onset form of Alzheimer disease [PMID: 16423463]
- polymorphism in the APP promoter correlates with late onset form of Alzheimer disease [PMID: 16243604]

- single nucleotide polymorphism in the APP promoter correlates with genetic predisposition to disease associated with Alzheimer disease [PMID: 17325276]

APOA1

- abnormal expression of APOA1 in cerebrospinal fluid correlates with Alzheimer disease [PMID: 14559363]
- abnormal folding of APOA1 may correlate with abnormal protein processing associated with Alzheimer disease [PMID: 7639323]
- decreased expression of APOA1 in serum correlates with increased severity of dementia associated with Alzheimer disease [PMID: 10794845]
- increased expression of APOA1 in cerebrospinal fluid correlates with Alzheimer disease [PMID: 10828089]

PPARG

- abnormal expression of PPARG protein may correlate with Alzheimer disease [PMID: 9920782]
- PPARG is expressed in brains of Alzheimer's Disease (AD) patients [PMID: 10685726]
- PPAR-gamma expression was selectively increased [PMID: 16873964]

Supplementary file 6

Network showing overlay of 43 literature-derived biomarkers on to the main AD network can be downloaded at the following URL:

http://www.scai.fraunhofer.de/de/geschaeftsfelder/bioinformatik/downloads.html

Network file in (.xml format) can be opened and visualized using Cytoscape_Version3.1.1.

To open the file with the Cytoscape; please follow these steps:

1. Download and install the appropriate Cytoscape version
2. Open Cytoscape
3. Please go to the file menu and select import Network(multiple file types) option.
4. Please browse through the downloaded network file.
5. Then select the network file and you will see the network in your cytoscape window.

Supplementary file 7

Statistical validation of network and knowledge cliffs

We generated 10,000 random networks from the original Alzheimer's disease network using R package "igraph". The function rewire.edges from the package was used to generate the random networks. The random networks were generated such that the power law of nodes in original network is preserved. From these random networks, we generated the probability of finding an edge at random as N/10,000, where N is the number of times an edge from the curated Alzheimer's network was found in the generated random networks. Assuming the independence of edges (i.e. edges are independent of one another), the probability of finding the network at random is product of probability of all the edges that is the p-value of the network. In the same way, the probability of a knowledge cliff in random network (p-value of the knowledge cliff) is just the product of all the edge probabilities in that cliff. Based on this we calculated the following value:

Knowledge cliff p-values
RTN4-BACE1-APP = 0.0409*0.0673 = 0.00275257

FBXO2-BACE1-APP = 0.0425*0.0673 = 0.00286025

F2LR3-BACE1-APP = 0.0353*0.0673 = 0.00237569

TP53INP2-BACE1-APP = 0.0369*0.0673 = 0.00248337

Network p-value: ~0
For 200 edges the combined p-value was 3.174614e-275, for 300 edges R package gives p-value as 0

The calculated numbers suggests that our results are significant and rejects null hypothesis for "random occurrence of knowledge cliffs"

The statistical analysis data along with probability values is available for download at the following URL:

http://www.scai.fraunhofer.de/de/geschaeftsfelder/bioinformatik/downloads.html
