# Supplementary material for: Exploring novel mechanistic insights in Alzheimer’s disease by assessing reliability of protein interactions
Source: Sci Rep. 2015 Sep 8;5:13634. doi: 10.1038/srep13634 (PMC4562155; doi:10.1038/srep13634)
Supplement: Supplementary dataset 2 [file srep13634-s3.doc]

# R functions to calculate protein-protein interaction scores

# usage:

# ppi_table <- read_table("/path/to/input.csv",header=TRUE,sep="\t",lastCol=22)

# ppi_scores <- compute_int_score(ppi_table)

# As per the instruction from the Manuscript administration, this R script is being uploaded in an alternative format. The authors would like to apologize for any inconvenience.

###############################################################################

###############################################################################

#

# Function: read_table : read files

# Parameters:

# input_file_path: /path/to/csv/file

# header: Boolean, if TRUE use first row as column names

# sep: character seperating entries in the given CSV file

# lastCol: column number of last column to read in (columns after lastCol are omitted)

# Return:

# data.frame

# sample entries:

# Protein1 Protein2 Directionality Relevence Pubmed id Date of Publication Journal Name Impact Factor FACT Fact/Hypo Invivo/Human MID Invivo/Animal MID Methods MID Primary Referential SCORE Final score Sentence

# A2M APP A2M-APP 1 12399113 2002 Brain Res Mol Brain Res 2.1 Fact Fact Immunoprecipitation,Western blot MI:0019,MI:0113 No Referential: 9202323 7,1 Alpha(2)M binds various cytokines, including IL-1beta, as well as Abeta.

# A2M APP A2M-APP 1 10679277 2000 Biochem Biophys Res Commun 2484 Fact Fact FPLC,Europium immunoassay MI:0091,MI:0678 Primary 7,1 Purified alpha2-macroglobulin was able to bind Abeta peptides and at physiological concentration bound 73% of 5 ng/ml of Abeta.

# TGF-_1 A2M TGF-_1-A2M 1 14678766 2003 Neurobiol Dis 5403 Fact Fact Nondenaturing PAGE analysis MI:0070 Primary 7,1 125I-A_ binding to native and methylamine-treated plasma _2M was determined by nondenaturing PAGE analysis

# A2M APP A2M-APP 1 9489740 1998 J Neurochem 4061 Fact Fact biochemical assay(protease activity) MI:0990 Primary 7,1 In the present study, we investigated whether alpha2-macroglobulin (alpha2M), a protein present in neuritic plaques and elevated in Alzheimers disease brain, is a potential regulatory factor for A beta fibril formation.

#

###############################################################################

read_table <- function(input_file_path,header=TRUE,sep=";",lastCol=22){

input_file <- file(input_file_path)

# trim files for unforseen errors

temp_list <- readLines(input_file)

temp_list <- gsub(pattern="(\\'|\\\")",replacement="",x=temp_list)

close(input_file)

temp_len <- c(1:length(temp_list))

line_list <- strsplit(temp_list,split=sep)

names(line_list) <- NULL

out_data <- matrix(NA,ncol=lastCol)

for(i in 1:length(line_list)){

if(length(line_list[[i]])>=lastCol){

temp_data <- line_list[[i]][1:lastCol]

if((nchar(temp_data[1])>=3)&&(nchar(temp_data[2])>=3)&&(nchar(temp_data[lastCol])>=3)){

temp_data <- gsub(pattern="(\\'|\\\")",replacement="",x=temp_data)

out_data <- rbind(out_data,temp_data)

}

}

}

out_data <- out_data[-1,]

rownames(out_data) <- NULL

if(header){

colnames(out_data) <- gsub(pattern="[^a-zA-Z0-9.]",replacement=".",x=out_data[1,],perl=TRUE)

out_data <- out_data[-1,]

}

clean_file <- as.data.frame(out_data,stringsAsFactors=FALSE)

clean_file <- clean_file[!apply(is.na(clean_file)|clean_file=="",1,all),c(1:lastCol)]

return(clean_file)

}

###############################################################################

#

# Function: compute_int_score : compute interaction score for the input data.frame

# Parameters:

# relevanceCol: column number for ppi interaction relevance

# journalCol: journal name column, nothing of significance, but to remove characters "\t|\\;") from the text

# impactCol: column number for journal imact factors

# factCol: column number for Fact/Hypothesis column

# invivoHuman_mid: column number for invivo human interaction detection method mids

# invivoAnimal_mid: column number for invivo animal interaction detection method mids

# invitroMethod_mid: column number for invtro method interaction detection method mids

# n_categories: number of rank divisions for the final score

# norm_method: MUST be one of "log" (default) OR "quantile" OR "uniform"

# : "quantile" OR "uniform" calls categorizer function for score to rank and rank to weight coversion

# Return:

# data.frame

#

###############################################################################

compute_int_score <- function(inputFrame,relevanceCol=4,journalCol=7,impactCol=8,factCol=10,invivoHuman_mid=12,invivoAnimal_mid=14,invitroMethod_mid=16,n_categories=15,norm_method="log",sort_out=FALSE){

inputFrame[,factCol] <- tolower(inputFrame[,factCol])

# get only the first one or two letters (-1|1) from relevanceCol

rel_col <- as.character(sub(pattern="^(\\-*1).*","\\1",inputFrame[,relevanceCol],perl=TRUE))

inputFrame[,relevanceCol] <- as.numeric(rel_col)

inputFrame[,impactCol] <- as.numeric(gsub(pattern=",",replacement=".",x=inputFrame[,impactCol]))

inputFrame[,journalCol] <- gsub(pattern=("\t|\\;"),replacement=" ",x=inputFrame[,journalCol],perl=TRUE)

inputFrame[,ncol(inputFrame)] <- gsub(pattern=("\t|\\;"),replacement=" ",x=inputFrame[,ncol(inputFrame)],perl=TRUE)

impFactor <- inputFrame[,impactCol]

impFactor[is.na(impFactor)] <- 0

final_score <- list()

for(c in 1:nrow(inputFrame)){

interaction_id <- paste(inputFrame[c,1],inputFrame[c,2],sep="-")

entry_score <- 0

fact <- FALSE

invivoHuman <- FALSE

invivoAnimal <- FALSE

invitro <- FALSE

invitro_mids <- character()

# check for fact

if(as.character(inputFrame[c,factCol])=="fact"){

fact <- TRUE

}

# check for invivo human/mouse

if(nchar(inputFrame[c,invivoHuman_mid])>3){

invivoHuman <- TRUE

}

if(nchar(inputFrame[c,invivoAnimal_mid])>3){

invivoAnimal <- TRUE

}

# get all the invitro methods and calculate aggregated score for an interaction

if(nchar(inputFrame[c,invitroMethod_mid])>3){

invitro <- TRUE

assays <- get_assays(as.character(inputFrame[c,invitroMethod_mid]))

invitro_mids <- method_checker(assay_vector = assays)

}

row_score <- calculate_score(fact = fact,invivoHuman = invivoHuman,invivoAnimal = invivoAnimal,invitro = invitro,invitro_mids = invitro_mids)

# orginal divisison, divide imapct factors into 12

imf_weight <- get_weight(inputFrame[c,impactCol])

row_score <- row_score + imf_weight

row_score <- row_score*as.numeric(inputFrame[c,relevanceCol])

if(is.null(final_score[[interaction_id]])){

final_score[[interaction_id]]$score <- row_score

}else{

final_score[[interaction_id]]$score <- final_score[[interaction_id]]$score + row_score

}

}

score_vec <-unlist(final_score)

names(score_vec) <- gsub(pattern="\\..*$",replacement="",x=names(score_vec))

if((norm_method=="quantile")||(norm_method=="uniform")){

out_data <- matrix(NA,nrow=1,ncol=ncol(inputFrame)+1)

colnames(out_data) <- NULL

for(r in 1:nrow(inputFrame)){

temp_frame <- inputFrame[r,]

interaction_id <- paste(inputFrame[r,1],inputFrame[r,2],sep="-")

interaction_scores <- score_vec[interaction_id]

temp_frame <- data.frame(temp_frame,interaction_scores)

colnames(out_data) <- colnames(temp_frame)

out_data <- rbind(out_data,temp_frame)

}

rownames(score_frame) <- NULL

# get score from categorizer function

cat_score<- categorizer(data=score_frame, Categories=n_categories, mode =norm_method , qlevels = NULL, inv=TRUE)

out_data2 <- matrix(NA,nrow=1,ncol=ncol(out_data)+2)

colnames(out_data2) <- c(colnames(out_data),"rank","rank_to_weight")

out_data <- out_data[-1,]

uniq_vec <- vector(mode="numeric",length=nrow(out_data))

for(rd in 1:nrow(out_data)){

tot_score <- out_data[rd,23]

for(r2 in 1:nrow(cat_score)){

score_frame_finalScore=as.numeric(cat_score[r2,1])

int_id <- paste(out_data[rd,1],out_data[rd,2],sep="-")

if((tot_score==score_frame_finalScore)&&(!is.element(rd,uniq_vec))){

temp_frame=data.frame(out_data[rd,],rank=cat_score[r2,2],rank_to_weight=cat_score[r2,3])

uniq_vec <- c(uniq_vec,rd)

out_data2 <- rbind(out_data2,temp_frame)

}

}

}

out_data2 <- out_data2[-1,]

if(sort_out){

out_data2 <- out_data2 [order(out_data2$rank_to_weight,decreasing=TRUE),]

}

rownames(out_data2) <- NULL

return(out_data2)

}else if(norm_method=="log"){

log_vec <- log10(score_vec+c)

norm_0_1 <- (log_vec-min(log_vec))/(max(log_vec)-min(log_vec))

score_frame <- data.frame(raw=score_vec,raw_100=(score_vec/100),log=log_vec,norm_0_1=norm_0_1)

rownames(score_frame) <- names(score_vec)

out_data <- matrix(NA,nrow=1,ncol=ncol(inputFrame)+ncol(score_frame))

colnames(out_data) <- c(colnames(inputFrame),colnames(score_frame))

for(r in 1:nrow(inputFrame)){

temp_frame <- as.data.frame(inputFrame[r,])

interaction_id <- paste(inputFrame[r,1],inputFrame[r,2],sep="-")

interaction_scores <- score_frame[interaction_id,1]

raw_by_100 <- score_frame[interaction_id,2]

log_score <- score_frame[interaction_id,3]

norm_score <- score_frame[interaction_id,4]

temp_frame <- data.frame(temp_frame,interaction_scores,raw_by_100,log_score,norm_score)

colnames(out_data) <- colnames(temp_frame)

out_data <- rbind(out_data,temp_frame)

}

out_data <- out_data[-1,]

out_data <- data.frame(out_data,norm_rounded=round(out_data$norm_score,digits=3))

if(sort_out){

out_data <- out_data [order(out_data$norm_rounded,decreasing=TRUE),]

}

rownames(out_data) <- NULL

return(out_data)

}else{

stop(" Could not recognize norm_method, parameter norm_method must be \"quantile\" OR \"uniform\" OR \"log\"! \n")

}

}

###############################################################################

#

# Function get_assays: internal method, clean input string and split into a vector on ","

# Parameter:

# inputString: string contataining method ids (mids), MUST BE comma separated

# Return:

# character vector

#

###############################################################################

get_assays <- function(inputString){

gsub(pattern="\\s{1,}",replacement="",x=inputString,perl=TRUE)

gsub(pattern="\\,{2,}",replacement="\\,",x=inputString,perl=TRUE)

assay_vec <- unlist(strsplit(x=inputString,split=","))

return(assay_vec)

}

###############################################################################

#

# Function method_checker: internal method, check assays and return classification

# Parameter:

# assay_vector: character vector of assay mids

# Return:

# character vector

#

###############################################################################

method_checker<- function(assay_vector){

library_based <- c("MI:0084","MI:0018","MI:0009","MI:0432","MI:0090")

genetic <- c("MI:0208","MI:0254")

insilico <- c("MI:0063")

# phys_chem <- c("MI:0019","MI:0055","MI:0012","MI:0872")

# if MI:0019 "Coimmunoprecipitation" and MI:0113 "Western blot" both are present then remove one

coimmuno <- "MI:0019"

wb <- "MI:0113"

if(is.element(coimmuno,assay_vector) && is.element(wb,assay_vector)){

assay_vector <- assay_vector[-which(assay_vector==wb)]

}

# return me

class_vec <- vector(mode = "character", length = length(assay_vector))

for(av in 1:length(assay_vector)){

if(is.element(assay_vector[av],library_based)){

class_vec[av] <- "lib"

}else if(is.element(assay_vector[av],genetic)){

class_vec[av] <- "gen"

}else if(is.element(assay_vector[av],insilico)){

class_vec[av] <- "ins"

}else{

class_vec[av] <- "phy"

}

}

return(class_vec)

}

###############################################################################

#

# Function calculate_score: given paramterters, calcualate interaction score

# Parameters:

# fact: Boolean (fact or Hypothesis)

# invivoHuman: Boolean (was invivo human method used ?)

# invivoAnimal: Boolean (was invivo animal method used?)

# invitro: Boolean (was invito methods used ?)

# invitro_mids: character vector containing invitro methods used

# Return:

# numeric value

#

###############################################################################

calculate_score <- function(fact,invivoHuman,invivoAnimal,invitro,invitro_mids){

row_score <- 0

# fact|hypo invivo Human

if(fact && invivoHuman){

row_score <- 100

}else if(!(fact) && invivoHuman){

row_score <- 65.33

}

# fact|hypo invivo Animal

if(fact && invivoAnimal){

row_score <- row_score+91.66

}else if(!(fact) && invivoAnimal){

row_score <- row_score+50

}

# fact|hypo invitro

if(fact&&invitro){

for(meth in 1:length(invitro_mids)){

if(invitro_mids[meth]=="lib"){

row_score <- row_score+61.16

}else if(invitro_mids[meth]=="gen"){

row_score <- row_score+66.66

}else if(invitro_mids[meth]=="ins"){

row_score <- row_score+39.16

}else if(invitro_mids[meth]=="phy"){

row_score <- row_score+81.16

}

}

}else if(!(fact) && invitro){

for(meth in 1:length(invitro_mids)){

if(invitro_mids[meth]=="lib"){

row_score <- row_score+23.33

}else if(invitro_mids[meth]=="gen"){

row_score <- row_score+25.33

}else if(invitro_mids[meth]=="ins"){

row_score <- row_score+8.33

}else if(invitro_mids[meth]=="phy"){

row_score <- row_score+38.91

}

}

}

return(row_score)

}

###############################################################################

#

# Function get_weight: Given an impact factor, use pre-determined impact factor

# divisions to convert the impact factor to weight

# Parameter:

# impact_factor: journal impact factor

# Return:

# numeric value

#

###############################################################################

get_weight <- function(impact_factor){

impact_factor <- as.numeric(impact_factor)

if(is.na(impact_factor)){

impact_factor <- 0

}

weight <- 0

n <- 12

if(impact_factor>0){

rank <- 0

if(impact_factor<=2.24){

rank <- 12

}else if(impact_factor<=2.66){

rank <- 11

}else if(impact_factor<=2.996){

rank <- 10

}else if(impact_factor<=3.58){

rank <- 9

}else if(impact_factor<=3.995){

rank <- 8

}else if(impact_factor<=4.061){

rank <- 7

}else if(impact_factor<=4.699){

rank <- 6

}else if(impact_factor<=5.103){

rank <- 5

}else if(impact_factor<=6.111){

rank <- 4

}else if(impact_factor<=7.584){

rank <- 3

}else if(impact_factor<=9.681){

rank <- 2

}else if(impact_factor>=10.264){

rank <- 1

}

weight <- (100*(n+(1-rank)))/n

}

return(weight)

}

##########################################################################

## Ranking function and Discretizer

## Author: Paurush Praveen

## Method: Uses quantile based discretization or uniform discretiztion

## to create desired number of categories in continuous variable vector.

## These levels can be used a ranks. The inverse of ranks is also

## supported via invRank function.

## Computes available categories in the data set via discretization

## Pre-requisite: R 2.15.x or above; platform independent,

## Library: No additional R libraries required

## Mail bug reports to: paurush@bit.uni-bonn.de

## 18 Nov 2013 10:03:18 AM CEST

##########################################################################

categorizer <- function(data, Categories, mode = "uniform", qlevels = NULL, inv=TRUE) {

numCategories=Categories

orig=data[,1]

if(!is.matrix(data) && !is.data.frame(data))

stop("data should be a matrix or data frame")

if(is.matrix(data)) {

samples <- data

outdataframe <- FALSE

}

else {

samples <- t(data)

outdataframe <- TRUE

}

numnodes <- dim(samples)[1]

numsamples <- dim(samples)[2]

if(numnodes < 1 || numsamples < 1)

stop("Invalid sample")

if(is.vector(numCategories)) {

len <- length(numCategories)

numcats <- floor(numCategories[len])

numcats <- rep(numcats, numnodes)

if(len <= numnodes) {

for(j in 1:len)

numcats[j] <- numCategories[j]

}

else {

for(j in 1:numnodes)

numcats[j] <- numCategories[j]

}

for(j in 1:numnodes)

if(numcats[j] < 2)

numcats[j] <- 2

}

else {

numcats <- floor(numCategories)

if(numcats < 2) {

warning("set numCategories to 2")

numcats <- 2

}

numcats <- rep(numcats, numnodes)

}

if(mode == "quantile") {

if(is.null(qlevels) || !is.list(qlevels) || length(qlevels) < numnodes) {

qlevels <- vector("list", numnodes)

for(j in 1:numnodes)

qlevels[[j]] <- seq(1:(numcats[j]-1))/numcats[j]

}

for(j in 1:numnodes) {

if(length(qlevels[[j]]) < numcats[j]-1 || min(qlevels[[j]]) < 0 || max(qlevels[[j]]) > 1)

qlevels[[j]] <- seq(1:(numcats[j]-1))/numcats[j]

}

}

if(mode == "uniform") {

if(is.null(qlevels) || !is.list(qlevels) || length(qlevels) < numnodes) {

qlevels <- vector("list", numnodes)

for(j in 1:numnodes)

qlevels[[j]] <- rep(1, numcats[j])

}

for(j in 1:numnodes) {

sl <- sum(qlevels[[j]])

if(length(qlevels[[j]]) < numcats[j] || sl <= 0) {

qlevels[[j]] <- rep(1, numcats[j])

sl <- sum(qlevels[[j]])

}

qlevels[[j]] <- qlevels[[j]]/sl

}

}

data <- matrix(rep(NA, length(samples)), nrow=dim(samples)[1])

for(j in 1:numnodes) {

col <- samples[j,]

ids <- !is.na(col)

col <- as.numeric(col)

if(!is.numeric(col))

stop("data should be numeric")

qq <- qlevels[[j]]

if(mode == "quantile") {

qq <- quantile(col[ids], qlevels[[j]])

}

if(mode == "uniform") {

if(sum(ids) > 0) {

minc <- min(col[ids])

maxc <- max(col[ids])

qq <- sapply(1:numcats[j], function(i) minc+(maxc-minc)*sum(qlevels[[j]][1:i]))

}

}

for(i in 1:length(col)) {

if(!ids[i])

next

id <- which(qq > col[i])

if(length(id)>0)

data[j,i] <- min(id)

else

data[j,i] <- numcats[j]

}

data[j,] <- as.integer(data[j,])

}

data <- matrix(as.integer(data), nrow=numnodes)

rownames(data) <- rownames(samples)

colnames(data) <- colnames(samples)

if(outdataframe) {

data <- as.data.frame(t(data))

## R converts integer back to numeric, hate it

for(j in 1:numnodes)

data[,j] <- data[,j]

}

mydata=data.frame(Original=orig, Ranks=data)

if(inv==TRUE){

mydata[,2]=invRank(mydata[,2])

}

# convert rank to weights

if(length(numCategories)==1){

weights <- (100*(numCategories+(1-mydata[,2])))/numCategories

mydata <- data.frame(mydata,Weights=weights)

mydata[which(mydata[,1]==0),3] <- 0

}

colnames(mydata)=c("Original", "Ranks","Weights")

return(mydata)

}

##########################################################################

## Ranking function and Discretizer

## Author: Paurush Praveen

## Inverts the ranks provoded by the ranker function

##########################################################################

invRank=function(categ){

maxV=max(as.numeric(categ))

newCateg=(maxV-as.numeric(categ))+1

return(newCateg)

}
